# Screening of novel therapeutic targets and chimeric vaccine construction against antibiotic-resistant *Yersinia Enterocolitica*

**DOI:** 10.3389/fimmu.2025.1555248

**Published:** 2025-07-04

**Authors:** Mohibullah Shah, Rouman Fatima, Asifa Sarfraz, Muhammad Umer Khan, Hasan Ejaz, Maqsood Alam, Shahid Aziz, Umar Nishan, Abid Ali, Ahmed Bari, Suvash Chandra Ojha

**Affiliations:** ^1^ Department of Biochemistry, Bahauddin Zakariya University, Multan, Pakistan; ^2^ Department of Animal Science, Federal University of Ceara, Fortaleza, Brazil; ^3^ Institute of Molecular Biology and Biotechnology, The University of Lahore, Lahore, Pakistan; ^4^ Department of Clinical Laboratory Sciences, College of Applied Medical Sciences, Jouf University, Sakaka, Saudi Arabia; ^5^ Department of Biochemistry and Molecular Biology, Federal University of Ceara, Fortaleza, Brazil; ^6^ Department of Chemistry, Kohat University of Science and Technology, Kohat, Pakistan; ^7^ Department of Zoology, Abdul Wali Khan University, Mardan, Pakistan; ^8^ Department of Pharmaceutical Chemistry, College of Pharmacy, King Saud University, Riyadh, Saudi Arabia; ^9^ Department of Infectious Diseases, The Affiliated Hospital of Southwest Medical University, Luzhou, China

**Keywords:** immunoinformatics, binding energy, antibody, antigenic, yersiniosis

## Abstract

*Yersinia enterocolitica* is known to cause a variety of infections, including mild gastroenteritis and severe systemic disease. This bacterium has developed resistance to several antibiotics, including cephalosporins, penicillins, and fluoroquinolones. Despite significant advances in vaccine formulation against *Y. enterocolitica*, there is no FDA-licensed vaccine available against it. Herein, the subtractive proteomics approach was utilized to determine the potential drug and vaccine targets, and then reverse vaccinology was utilized to formulate effective vaccines against this pathogen. A core proteome was constructed from the available 22 complete genomes of *Y. enterocolitica*. Screening resulted in 14 non-human homologous, essential, and virulent proteins being identified as drug targets, while 15 were identified as vaccine targets. The predicted vaccine targets were analyzed, and as a result, two proteins met the criteria for epitope prediction. The epitopes were subjected to a screening pipeline to identify epitopes capable of inducing both T- and B-cell-mediated immune responses. Four vaccine constructs were designed using the selected epitopes by adding the appropriate adjuvants and linkers. The chosen T-cell epitopes showed the possibility of covering 99.26% of the global population. The constructs V1, V2, V3, and V4 were top-ranked based on their physicochemical properties and selected for further analysis. These four vaccines were computationally docked with immune receptors TLR4 and TLR5 to evaluate binding affinities, with V2 and V4 displaying the highest binding affinities with TLR4. The MD simulations, NMA, binding free energy, PCA, and DCCM analysis ensured the stability of complexes. Immune simulations predicted a high immunological profile for the V2 and V4 constructs. Furthermore, *in-silico* cloning assured that the proposed vaccines could be efficiently expressed in the *E. coli* (K12) vector. This study provides valuable insights into developing effective vaccines against *Y. enterocolitica*; however, the immunogenicity of the designed vaccine requires experimental validation.

## 1 Introduction

*Yersinia enterocolitica*, belonging to the genus *Yersinia*, family *Enterobacteriaceae*, is a gram-negative, bacillus-shaped bacterium that causes gastrointestinal disease in humans (1). The first reported case of *Y. enterocolitica* infection in humans was in 1939 when it was initially identified as Bacterium entercoliticum. In 1963, it was temporarily renamed Pasteurella X, and in 1964, it was reclassified under the genus *Yersinia* (2). *Yersiniosis* is a widely documented zoonotic gastroenteritis (3) caused by *Y. enterocolitica* and is primarily transmitted via the fecal-oral route. *Y. enterocolitica* has been detected in both domestic and wild animals, including goats, dogs, cats, sheep, cattle, and, most notably, pigs. Pigs are regarded as the principal carriers of human-transmissible pathogenic strains due to their great prevalence and genetic similarities. Although direct contact with pets and polluted water are the risk factors for the spread of this pathogen, the predominant method of transmission is the ingestion of raw or uncooked pork. Furthermore, flies found in pig farms and kitchens may play a role in its transmission from animals to humans, underlying their potential as arthropod vectors (4, 5). Notably, flies in Libyan hospitals have been found carrying antibiotic-resistant strains of *Enterobacteriaceae*, potentially facilitating nosocomial infection (6). In addition to causing diarrhea and abdominal pain, yersiniosis may lead to extraintestinal problems like erythema nodosum, conjunctivitis, and arthritis, often with symptoms resembling appendicitis in children and young adults (7). *Y. enterocolitica* initially replicates in Peyer’s patches before spreading to mesenteric lymph nodes, spleen, and liver, where they form monoclonal micro abscesses (8). Enteric Yersinia optimally produces invasion proteins encoded by the chromosomal *y* locus in conditions of lower ambient temperatures and acidic environments similar to those present within the host (9). Upon ingestion, invasion binds to B1 integrins on host cells, allowing epithelial penetration. Following a temperature increase within the host, the expression of virulence proteins, which are essential for lymph tissue colonization and immune invasion, increases (10).

Yersiniosis is the fourth most common bacterial gastrointestinal disease in Europe, and a significant decrease in incidence has been seen over the previous five years (11). However, there are currently no effective treatments for infections caused by this bacterium. Although many environmental and foodborne *Y. enterocolitica* strains are non-pathogenic or weakly pathogenic, certain pathogenic *Y. enterocolitica* strains, especially serogroups O:3, O:9, O:8, and O:5,27, are commonly linked to human disease and harbor both chromosomal and plasmid-encoded virulence factors (12). Notably, highly virulent biotypes like 1B/O:8 and 4/O:3 have been associated with severe infections and global outbreaks (13). Most *Y. enterocolitica* strains are resistant to trimethoprim-sulfamethoxazole, tetracyclines, aminoglycosides, fluoroquinolones, penicillin, ticarcillin, cefazolin, cephalothin, clavulanate, and ampicillin, and first- and third-generation cephalosporins (14). Moreover, the European Committee on Antimicrobial Susceptibility Testing (EUCAST) has recognized *Y. enterocolitica’s* intrinsic resistance to cefoxitin and cefamandole, further highlighting its antibiotic resistance profile (15). Despite its clinical relevance, *Y. enterocolitica* remains understudied in vaccine and therapeutic development. The emergence of extremely virulent and multidrug-resistant *Y. enterocolitic*a strains in recent years is concerning for public health, necessitating the identification of novel therapeutic targets. This study fills that gap by finding novel therapeutic targets and developing a multi-epitope vaccine, intending to improve public health outcomes by controlling the antibiotic-resistant *Y. enterocolitica*. Vaccination could serve as a preventive measure, particularly in regions with frequent outbreaks or where sanitation practices are inadequate.

Vaccination is an important and cost-effective technique for controlling infectious illnesses, helping individuals and communities alike by lowering illness and healthcare expenditures (16). Traditional vaccines rely on cultivating and processing microorganisms to elicit immune responses, but these procedures are frequently time-consuming, expensive, and ineffective against specific infections (17). Advances in vaccine development now use genetic and proteomic data to construct peptide-based subunit vaccines that target antigenic protein areas to improve efficacy and specificity via multi-epitope methods (18). Currently, no licensed vaccine exists to prevent *Y. enterocolitica* infections. This emphasizes the need to find potential vaccine candidate proteins and validate their efficacy in animal models. Traditional vaccine development is often limited by hypersensitivity risks, poor attenuation, low immunogenicity, and high costs, emphasizing the need for modern approaches like *in-silico* vaccine design (19, 20).

In this study, subtractive proteomics was used on the core proteome of *Y. enterocolitica*, and multiple drug and vaccine targets were identified. The reverse vaccinology was then applied to the identified vaccine targets to develop multi-epitope peptide vaccines against *Y. enterocolitica* that include both linkers, B- and T-cell epitopes, and adjuvants. The designed vaccine candidates revealed the ability to trigger powerful immune responses. The final vaccines were successfully reverse-translated and expressed in *E. coli* (K12 strain) via *in silico* cloning. The peptide-based vaccine designed in this study against *Y. enterocolitica* demonstrates strong immunological potential and represents a promising candidate for further experimental validation to confirm its efficacy and safety.

## 2 Materials and methods

**Figure 1:**
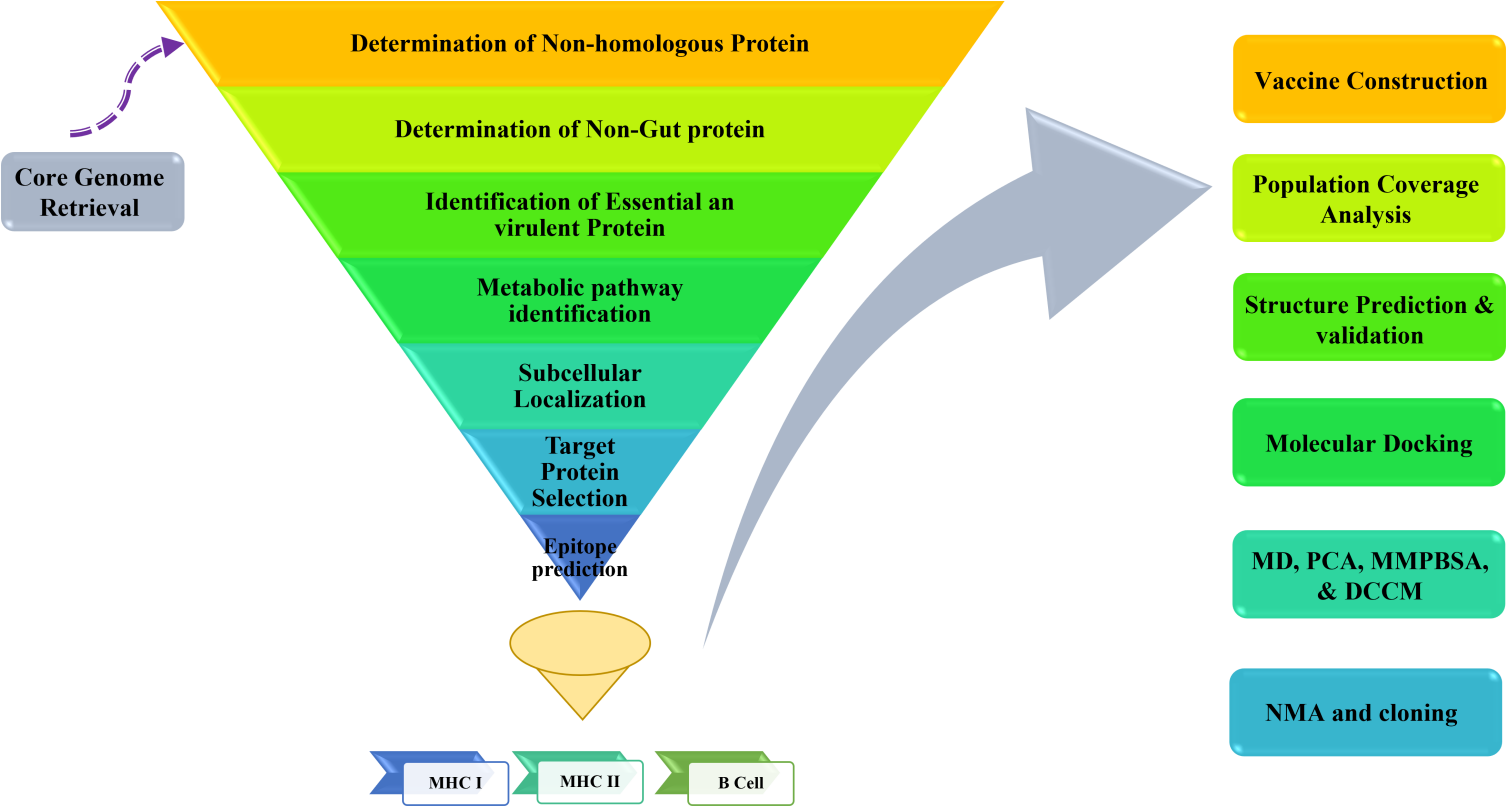
A template of the process for selecting and verifying potential immunogenic targets and designing a Multi-Epitope Vaccine (MEV) for *Y. enterocolitica* using advanced bioinformatics tools and reverse vaccinology methods.

Subtractive proteomics and reverse vaccinology were utilized to discover possible therapeutic targets for *Y. enterocolitica* and develop an immunogenic vaccine. The approach used in the investigation is shown in **Figure 1**.

### 2.1 Data retrieval

The core proteome of 22 complete *Y. enterocolitica* genomes was acquired using the EDGAR database (21). Both resistant and non-resistant strains were chosen to ensure the vaccine’s effectiveness against all strains. The human proteome data was downloaded from the UniProt database for comparative study (22).

### 2.2 Host non-homologous protein identification

BLASTp analysis was performed to detect host non-homologous proteins with threshold values of bitscore < 100, identity < 35%, qcovs < 35%, and E-value >1e-20 (23, 24). Proteins showing similarities with human proteins were excluded. The shortlisted proteins were analyzed by BLASTp against the human gut microbiome with the same cutoffs to ensure that they are non-homologous to the human gut microbiome proteins (25, 26).

### 2.3 Identification of pathogen-essential and virulent proteins

The pathogen’s essential proteins were identified using the Database of Essential Genes (DEG) (27). DEG comprises all necessary genes for pathogen survival. To identify important genes in *Y. enterocolitica*, BLASTp analysis was performed against DEG with cutoff criteria of the query coverage > 35%, bit-score > 100, % identity > 35%, and E-value < 1e-20 (28). Moreover, bacterial virulence destroys the host’s defense mechanism and contributes to disease transmission via invasion, colonization, and adhesion. The virulence factor database (VFDB) identifies four kinds of virulence factors in pathogens, such as aggressive, defensive, non-specific, and virulence-related proteins. Pathogen virulent proteins were identified using BLASTp against VFDB with similar cutoffs as DEG.

### 2.4 Analysis of metabolic pathway

The KEGG database was utilized to investigate proteins involved in the unique *Y*. *enterocolitica* metabolic pathways. Following the proteins from the previous step, the KAAS server was used to assign KO (Kegg-Orthology) numbers to the proteins. Furthermore, the pathways of humans and the pathogen were determined by the KEGG pathway tool (29). In order to determine the pathogen’s unique metabolic pathways, pathogens and human host metabolic pathways were compared manually, and then proteins from unique and common pathways were identified.

### 2.5 Subcellular localization

All proteins require a precise place to function optimally. Determination of the subcellular localization of the proteins is important for the prediction of appropriate drug and vaccine targets. The PSORTb 3.0 (30) was used for the identification of subcellular localization of proteins, and CELLO version 2.5 (31) was used for further confirmation (22). The membrane and extracellular proteins were chosen as potential vaccine targets, while the cytoplasmic and periplasmic proteins were predicted as drug targets.

### 2.6 Selection of vaccine protein

The identified vaccine target proteins were evaluated for their potential to be vaccine candidates. The VaxiJen v2.0 server (32) was utilized to assess antigenicity, while the AllergenFp server (33)determined allergenicity. The physicochemical properties, water solubility, and topology value, were assessed using the ProtParam Expasy server (34), TMHMM (35), and SolPro servers, respectively. Highly antigenic and non-allergenic proteins were identified as potential vaccine candidates.

### 2.7 Prediction and evaluation of T cell epitopes

The prediction of epitopes is an important step in developing multi-epitope vaccines. Protein epitopes can elicit immunological responses from both T- and B-cells. The cellular immune response consists of two types of T-cells: cytotoxic T-cells and helper T-cells, which detect epitopes presented by MHC molecules, specifically MHC-I and MHC-II. The IEDB (Immune Epitope Database) was utilized for the prediction of MHC-binding epitopes (36). When designing vaccines, combining binding affinity predictions with ligand elution data ensures a more comprehensive selection of immunogenic epitopes. Therefore, the NetMHCpan 4.1 EL tool in the IEDB, which utilize both types of data to improve the prediction of peptide-MHC interactions, was used to predict cytotoxic T-lymphocyte (CTL) and helper T-lymphocyte (HTL) in the identified proteins. Epitope lengths of 9–10 amino acids for CTL and 15 amino acids for HTL epitopes were deemed ideal. The top epitopes with the lowest rank from each category were then tested for allergenicity, antigenicity, and toxicity using AllergenFp (33), VaxiJen v2.0, and ToxinPred (37), respectively. The IL4pred (38), IL10pred (39), and IFNepitope (40) tools were used to assess the interferon-gamma and interleukin-10, including the potential of MHC-II binding epitopes.

### 2.8 Identification and analysis of B-cell epitopes

B-cell epitopes are the regions of an antigen surface that particular antibodies detect and attach to, and induce an immunological response (41). Therefore, B-cell epitopes are anticipated to be helpful in the development of vaccines. The BepiPred Linear Epitope Prediction 2.0 (42) of IEDB-AR was employed to identify linear B-cell epitopes. Only epitopes with lengths ranging from 10 to 40 were chosen and investigated further. These B-cell epitopes were analyzed by utilizing different servers, i.e., AllergenFp (allergenicity), ToxinPred (toxicity), Innovagen (water solubility), and Vaxijen 2.0 (antigenicity).

### 2.9 Chimeric vaccine construction

To create chimeric vaccine constructs, the prioritized MHC-1, MHC-2, and B-cell epitopes were joined together using amino acid linkers, specifically EAAAK, AAY, GPGPG, and KK. Linkers are essential for connecting epitopes so that vaccines can work properly (43). The EAAAK linker was attached to the N and C terminals to provide rigidity to the ends. The AAY linker was used to join MHC-I epitopes while the GPGPG linker was used to join MHC-II epitopes, and the KK linker was used to join epitopes of B-cells. Adjuvants are required to reduce the antigenic material dose, resulting in robust immune responses. Four adjuvants, i.e., beta-defensin, HBHA conserved, flagellin, and L7/L12 ribosomal protein sequences, were combined to create four different vaccine constructs (44).

### 2.10 Vaccine construct analysis

Numerous criteria were utilized to evaluate the vaccine construct after the creation of the vaccine design, like the Vaxijen v2.0 server, which was used for antigenicity with a ≥ 0.4 cutoff value. Similarly, the Protparam server was used for molecular weight, gravy value, instability index, aliphatic index, total number of amino acids, and theoretical pi of protein vaccine design (45). Topology value and allergenicity were calculated by using TMHMM and AllerTop servers, respectively. The molecular weight impacts antigenicity, the theoretical Pi indicates a vaccine’s hydrophilic and hydrophobic nature, the aliphatic index ensures thermostability, the instability index provides information about vaccine construct stability, and the hydropathy index (GRAVY value) represents vaccine construct polarity.

### 2.11 Analysis of post-translational modification

Post-translational modifications (PTMs) are the enzymatic modifications that impact protein activity, structures, and dynamics (46) PTMs of the designed vaccine constructs were analyzed. NetPhos 3.1 was used to predict phosphorylation sites in the protein sequence (47) and NetNGlyc 1.0 was utilized to identify N-Glycosylation sites (48).

### 2.12 Prediction of secondary and tertiary structures

The SOPMA server (49) was used for the prediction of the secondary structure of the vaccine constructs, generating alpha-helices, beta-turns, random coils, and extended strand structures within the constructs. The GlobPlot was used to determine both globular and disordered regions. Protein’s tertiary structure is crucial for molecular function; therefore, the 3D structures of the vaccines were determined using the trRosetta server (50).

### 2.13 Validation of tertiary structure

The Ramachandran plot can be used to validate the protein models, whether they are computationally predicted or empirically determined. More than 90% of the residues in protein structures located in favorable regions indicate the good quality of the models (44). The PROCHECK server was used to confirm the 3D structures with Ramachandran plot analysis. Furthermore, the ERRAT plot, which assesses non-bonded atomic interactions, was used to establish protein structural quality. This method is very effective for tracking the progression of crystallographic refining and modeling. A higher ERRAT score indicates better protein model quality, as predicted by the SAVES server (51).

### 2.14 Population coverage analysis

The population coverage tool from the IEDB database (36) was used to evaluate coverage of T-cell epitopes worldwide, ensuring that the vaccine’s epitopes match the MHC-binding alleles distributed among different groups. This was crucial since *Y. enterocolitica* impacts people worldwide, and the goal was to achieve broad vaccine efficacy (52).

### 2.15 Molecular docking

The binding affinities of the designed vaccine constructs to host immunological receptors, TLR-4 (PDB ID: 4G8A) and TLR-5 (PDB ID: 3JOA), were assessed using ClusPro server. The vaccine constructs with the highest binding energy and the most H-bond interactions with the receptor were chosen for further investigation. PyMOL was used to quantify the bond lengths between the vaccine and receptor, which are important for describing molecular interactions. It enables exact measurement of bond lengths, resulting in deep insights into molecular interactions (53).

### 2.16 Normal mode analysis

To determine the stability of the complex with the number of contacts and highest binding affinity, NMA evaluation was used. The iMODs server was used to execute the NMA of the top-docked complex and assess its structural stability (54). This service predicted NMA in internal coordinates, allowing for the investigation of collective macromolecules such as proteins. Key characteristics such as eigenvalues, variance, B-factors, covariance, deformability, and the elastic network of the docked complex were examined, and the findings were shown graphically (55).

### 2.17 MD simulation

Molecular dynamic simulations of the vaccine-receptor docked complexes were performed to ensure their stability, flexibility, and the interaction of the vaccine within the biological framework. The AMBER 21 was utilized for these simulations and energy optimization (56). MD simulations were used to mimic the docked complexes’ natural biological environment. A 10.0 Å TIP3P box and FF19SB force field were used for the system solvation, while the Na^+^ counter ions were added to neutralize the system. Two minimization rounds were run with 3000 & 6000 steps to reduce the adverse collisions. After that, the system was equilibrated at 1 ATM pressure and heated to 300 K.

#### 2.17.1 Analysis of MD trajectories

The CPPTRAJ module and PTRAJ tools from the AMBER 20 software package were used to examine the stability, folding, and flexibility of the proposed proteins of each system’s 100-nanosecond-long output trajectory of the vaccine construct-TLR complex. The simulation was completed in three steps: system setup, trajectory analysis, and pre-processing. For docked complexes, compactness is essential for maximum stability (57). During the trajectory analysis phase, five trajectories—”Root Mean Square Deviation (RMSD),” “Root Mean Square Fluctuations (RMSF),” “Solvent Accessible Surface Area (SASA),” “Radius of Gyration (Rg),” were developed to assess structural changes, simulation stability, protein stability during the simulations for the vaccine-receptor complexes, the docked complex compactness and relaxation, and the identification of potent immunogenic regions and stability of complex, respectively.

#### 2.17.2 Binding free energy calculation

Binding free energies were estimated using snapshots acquired during MD simulation to investigate each complex’s energetic and structural properties. To determine binding free energy, molecular mechanics-based analysis was performed, with molecular mechanics MMPBSA modules integrated into the AMBER 21 software (58). For MMPBSA computations, 1000 snapshots were computed for each complex system using the current two-ns MD trajectories. The AMBER MMPBSA is a basic method for determining the binding free energy of ligands bound to receptors. Equation 1 was employed to predict the MMPBSA binding free energy:


(1) $$\Delta G_{bind}=\Delta G_{receptor\;+\;Lignad}-\left(\Delta G_{Recetor}+\Delta G_{Ligand}\right)$$

To compute net binding free energy (ΔG) in a system, subtract the total binding energy of the receptor and ligand from the complex. The gas phase energy change, ΔG_gas_, is computed using molecular mechanics, which comprises van der Waals and electrostatic energy. The solvation-free energy change (ΔG_solv_) consists of both nonpolar and polar energy components (Equation 2). Molecular mechanics energy fragmentation was further analyzed as non-bonded electrostatic energies (E_ele_), vdW energies (E_vdW_), and the solvation free energy (G_sol_), which was further divided into nonpolar and polar solvation energies, respectively.


(2) $$\Delta G_{sol}=\Delta G_{ele,solPB(GB)}+\Delta G_{nonpol,sol}$$

To establish the disintegration parameters of the polar (G_ele,sol_), nonpolar (G_nonpol,sol_), electrostatic (G_ele_), and vdW (E_vdW_) energies, binding free energies were further split down into different residual contributions for vaccination and TLR4 receptor interaction, as shown in Equation 3. The binding free energy snapshots were also utilized to evaluate the breakdown parameters.


(3) $$\Delta G_{vaccine–\;receptor}=\Delta G_{vdW}+\Delta G_{ele}+\Delta G_{ele,sol}+\Delta G_{nonpol,sol}$$

MMPBSA computations provide vital information on the molecular processes underlying ligand binding and may aid in the creation of new vaccines with better efficiency and specificity.

#### 2.17.3 PCA analysis and Gibb’s free energy analysis

The principal component analysis (PCA) (59) was used to determine the primary routes of motion in each complex. The most important key elements, 1 (PC1) and 2 (PC2), were analyzed using the AMBER 21 packages (60). This statistical method analyzes the covariance matrix of alpha-carbon (Cα) atoms to decrease and identify major modifications in the protein. It estimates the essential elements of the protein and provides details regarding its dynamics. The Gibbs free energy landscape (Gibbs FEL) analysis is utilized to analyze the stability of the TLR-vaccine complexes. Gibbs free energy was calculated using two main elements (PC1 and PC2). The stability of the system and its corresponding configuration diminish the Gibbs free energy value (61).

#### 2.17.4 Dynamic cross-correlation matrix

During a 100 ns molecular dynamics (MD) simulation, the dynamic cross-correlation matrix (DCCM) was utilized to investigate the correlated motions of residues in bound docked complexes. The cross-correlation coefficients between the variations of each pair of residues were computed by analyzing the trajectory data from the MD simulation. The resulting DCCM was displayed as a heatmap, with colors ranging from -0.6 (anti-correlated movements) to 1.0 (positively correlated motions). This approach revealed the dynamic behavior and potential functional connections of vaccine-receptor-docked complexes, allowing the identification of locations within the complex with noticeable correlated or anti-correlated motions.

### 2.18 Immune simulations

To evaluate the vaccine’s efficacy and immune profile, computational immune simulations were executed using the C-ImmSim server. Simulation parameters included 1000 steps, a simulation volume of 10, random speed sets to 12345, and injection points at time steps of 1, 84, and 168 hr. All other parameters were maintained at their default settings to model the immune response effectively (62, 63).

### 2.19 Conformational B-cell epitope prediction

Conformational B-cell epitopes are made up of numerous discontinuous amino acid sequences that, while separated in the original sequence, come together in the folded protein structure to generate binding sites for antibodies (64). The Ellipro server was used to anticipate and assess conformational B-cell epitopes in the designed vaccine (65).

### 2.20 Codon optimization and in silico cloning

The vaccine’s constructs codons were optimized with the Jcat server in *E. coli* (K12 strain) (66). Codon optimization aimed to maximize the vaccine protein’s expression efficiency and yield in the host organism by analyzing CAI value, codon usage bias markers, and GC content while ensuring the correct amino acid sequence. The optimal CAI value is 1.0, while the optimal GC content ranges from 30 to 70%. Optimized codon sequences were cloned using SnapGene software (67) into the pET-28a+ plasmid vector for expression in the host organism (68).

## 3 Results

### 3.1 Determination of human host non-homologous proteins

A core genome was formulated based on 22 publicly available complete genomes of *Y. enterocolitica*. This dataset encompassed the multidrug-resistant strains and a range of serotypes, including those associated with human pathogenicity, such as O:3, O:8, and O:9. The *Y. enterocolitica* core genome contained 57409 proteins. After removing duplicate proteins, 2483 non-paralogous core proteins were found. These proteins were filtered out to remove the human homologs to reduce the possibility of adverse consequences. After the human proteome and gut microbiome blast, 192 human non-homologous proteins were determined, which were proceeded further.

### 3.2 Determination of pathogen essential and virulent proteins

Essential genes encode proteins that are required for fundamental cellular activities, making them important targets in drug and vaccine development (69). The proteins shortlisted from the prior step were examined using BLASTp against the DEG, resulting in the identification of 67 essential proteins. Additionally, the identification of virulence factors was necessary, as they play a crucial role in the bacterial involvement in various diseases. BLASTp analysis against the VFDB identified 19 virulence-associated proteins of *Y. enterocolitica*. These proteins were combined, and 6 duplicates were removed, resulting in 80 essential and virulent proteins, which were proceeded further.

### 3.3 Metabolic pathway analysis

The KEGG (Kyoto Encyclopedia of Genes and Genomes) server provided metabolic pathways for both *Y. enterocolitica* (123 pathways) and the human host (356 pathways). The metabolic pathways of *Y.* enterocolitica and humans were analyzed to discover their common and unique pathways. The resulting common pathways between *Y.* enterocolitica and humans were 162, while 42 pathways were unique (**Table 1**). 80 pathogen-important proteins discovered using comparative subtractive genomics were functionally annotated utilizing the KAAS service (KO assignment) via metabolic pathway analysis. 72 proteins were found to be KEGG-dependent and engaged in pathogen-unique metabolic pathways, and 8 proteins were classified as KEGG-independent. Out of 72 KEGG-dependent proteins, 21 proteins were mapped in pathogen-unique pathways. These 21 mapped KEGG-dependent and 8 independent proteins were subjected to subcellular localization.

**Table 1:** Unique metabolic pathways of *Yersinia enterocolitica*.

Sr. no	Entry	Name
1.	yel00261	Monobactam biosynthesis
2.	yel00300	Lysine biosynthesis
3.	yel00332	Carbapenem biosynthesis
4.	yel00361	Chlorocyclohexane and chlorobenzene degradation
5.	yel00362	Benzoate degradation
6.	yel00364	Fluorobenzoate degradation
7.	yel00401	Novobiocin biosynthesis
8.	yel00460	Cyanoamino acid metabolism
9.	yel00521	Streptomycin biosynthesis
10.	yel00523	Polyketide sugar unit biosynthesis
11.	yel00525	Acarbose and validamycin biosynthesis
12.	yel00540	Lipopolysaccharide biosynthesis
13.	yel00541	O-Antigen nucleotide sugar biosynthesis
14.	yel00542	O-Antigen repeat unit biosynthesis
15.	yel00543	Exopolysaccharide biosynthesis
16.	yel00550	Peptidoglycan biosynthesis
17.	yel00552	Teichoic acid biosynthesis
18.	yel00623	Toluene degradation
19.	yel00625	Chloroalkane and chloroalkene degradation
20.	yel00626	Naphthalene degradation
21.	yel00627	Aminobenzoate degradation
22.	yel00633	Nitrotoluene degradation
23.	yel00643	Styrene degradation
24.	yel00660	C5-Branched dibasic acid metabolism
25.	yel00680	Methane metabolism
26.	yel00907	Pinene, camphor and geraniol degradation
27.	yel00930	Caprolactam degradation
28.	yel00998	Biosynthesis of various antibiotics
29.	yel00999	Biosynthesis of various plant secondary metabolites
30.	yel01053	Biosynthesis of siderophore group nonribosomal peptides
31.	yel01110	Biosynthesis of secondary metabolites
32.	yel01120	Microbial metabolism in diverse environments
33.	yel01220	Degradation of aromatic compounds
34.	yel01501	beta-Lactam resistance
35.	yel01502	Vancomycin resistance
36.	yel01503	Cationic antimicrobial peptide (CAMP) resistance
37.	yel02020	Two-component system
38.	yel02024	Quorum sensing
39.	yel02030	Bacterial chemotaxis
40.	yel02040	Flagellar assembly
41.	yel02060	Phosphotransferase system (PTS)
42.	yel03070	Bacterial secretion system

### 3.4 Subcellular localization

Establishing the subcellular positioning of a protein is crucial for comprehending its function in diseases and for creating new drug and vaccine targets. Out of 29 (8 KEGG-independent and 21 KEGG-dependent), 14 proteins were located in the cytoplasm and periplasm, so they were predicted as drug targets, whereas 1 was extracellular, and 14 were membrane proteins, such as outer membrane and plasma membrane, so collectively these 15 proteins act as vaccine targets (**Table 2**).

**Table 2:** Subcellular localization of vaccine proteins.

Protein accession no.	Psortb results	Cello results	BUSCA results	Final localization
WP_046050024.1	Cytoplasmic membrane	Inner membrane or Cytoplasmic	Cytoplasmic	Membrane protein
WP_050163389.1	Extracellular	Extracellular	Cytoplasmic	Extracellular
WP_019080857.1	Flagellar	Inner membrane	Plasma membrane	Membrane Protein
WP_263696491.1	Cytoplasmic membrane	Inner membrane	Plasma membrane	Membrane protein
WP_019084175.1	Cytoplasmic membrane	Inner membrane	Plasma membrane	Membrane protein
WP_019082060.1	Cytoplasmic membrane	Inner membrane	Plasma membrane	Membrane protein
WP_019080861.1	Cytoplasmic membrane	Inner membrane	Plasma membrane	Membrane protein
WP_046051349.1	Cytoplasmic membrane	Inner membrane	Plasma membrane	Membrane protein
WP_019079071.1	Cytoplasmic membrane	Periplasmic or Cytoplasmic	Extracellular space	Membrane protein
WP_050157306.1	Outer membrane	Outer membrane	Extracellular space	Outer membrane
WP_005164390.1	Cytoplasmic membrane	Inner membrane	Plasma membrane	Membrane protein
WP_050161901.1	Outer membrane	Outer membrane	Extracellular space	Outer membrane
WP_019079224.1	Outer membrane	Outer membrane	Outer membrane	Outer membrane
WP_019083129.1	Cytoplasmic membrane	Periplasmic	Plasma membrane	Membrane protein
WP_050131380.1	Cytoplasmic membrane	Inner membrane	Plasma membrane	Membrane protein

### 3.5 Target protein identification

Target proteins were selected based on their antigenic and non-allergenic properties, predicted using the AllergenFp and VaxiJen v2.0 server. The Protparam Expasy server was utilized to predict the physicochemical properties of selected proteins, like theoretical pi, molecular weight, instability index, query length, aliphatic index, Gravy value, and half-life. The TMHMM server identified proteins with topology 0 or 1 by analyzing transmembrane helices in their sequences. (**Supplementary Table S1**). Among the 15 vaccine target proteins, three fulfilled the criteria, of which two proteins (WP_019079224.1 and WP_050161901.1) were selected as potential vaccine candidates.

### 3.6 T-cell epitopes prediction

The T-cell epitopes were identified using the NetMHCpan EL 4.0 web server, recommended by IEDB. For epitope prediction, the complete HLA reference allele dataset from IEDB was chosen. For protein (WP_019079224.1), the server produced 18981 MHC-I binding epitopes, while for WP_050161901.1, it generated 40797. The epitopes with a rank less than 0.9 were chosen for further analysis. The Vaxijen v2.0, Toxinpred, and AllergenFp servers were used to examine the top 20 epitopes with the lowest score from both proteins for allergenicity, antigenicity, and toxicity, respectively. Furthermore, MHC-I binding epitopes’ immunogenicity was assessed using the IEDB Class-I immunogenicity prediction method. For vaccine construction, only 1 MHC-I epitope from WP_050161901.1 and 1 from WP019079224.1 were chosen due to their non-toxicity, non-allergenicity, and antigenicity (**Supplementary Tables S2**, **S3**).

The server produced 2422 MHC-II epitopes for WP_019079224.1 and 5250 for WP-050161901.1 protein. The epitopes with a rank less than 0.9 were chosen for further analysis. The toxicity, antigenicity, and allergenicity prediction of the top 20 epitopes of both proteins with the lowest rank were examined. Furthermore, IL4-Pred, IL10-Pred, and IFN epitope servers were utilized to analyze the IL-4, IL-10, and IFN-gamma-inducing characteristics of MHC-II binding epitopes, respectively. After analysis, 3 epitopes were selected from the WP_050161901.1 protein and 3 from WP_019079224.1 based on their non-allergenic, antigenic, non-toxin, and cytokine induction properties, so they were chosen for the construction of the vaccine (**Supplementary Tables S4**, **S5**).

### 3.7 Prediction of B-cell epitopes

B-cell epitopes are crucial for designing multi-epitope vaccines because they stimulate immune responses by attaching to B-cell receptors and producing antibodies. 9 epitopes were identified for WP_019079224.1 and 16 epitopes for WP_050161901.1 by utilizing BepiPred Linear Epitope prediction server 2.0 of IEDB-AR. From these, epitopes with a preferred length of 10–40 amino acids were selected, resulting in 4 epitopes from WP_019079224.1 and 8 epitopes from WP_050161901.1 being chosen for further analysis (**Supplementary Tables S6**, **S7**).

### 3.8 Chimeric vaccine construction and analysis

**Figure 2:**
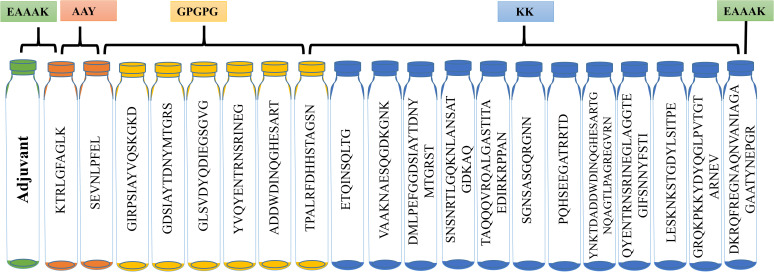
Vaccine construct design by sequential combination of MHC-I, MHC-II, and B-cell epitopes via AAY, GPGPG, and KK linkers in between them. Adjuvant is attached at the N-terminal.

The lead T- and B-cell epitopes from previous analyses were used for vaccine construction. Linkers such as EAAAK, GPGPG, AAY, and KK linked the epitopes. The antigenicity and allergenicity of the constructs, as influenced by adjuvant inclusion, were evaluated using various adjuvant combinations. The inclusion of linkers did not change the conformation of the planned structures. Beta-defensin, flagellin, HBHA conserved adjuvant, and L7/L12 ribosomal proteins were integrated at the N-terminal of the selected epitopes to boost immunogenicity (**Figure 2**). Ultimately, the combined lead epitopes, adjuvants, and linkers produced four unique vaccine constructs: V1, V2, V3, and V4. The AntigenPro server used a cutoff value of >0.90 to evaluate vaccine component antigenicity. The VaxiJen server was used to determine antigenicity, and a limit of 0.75 indicates good antigenic behavior. The ProtParam server assessed molecular weight, aliphatic index, instability index, gravy value, number of amino acids, and theoretical pi. The generated constructs were analyzed for solubility using the SolPro server. Topology value and allergenicity were determined by using TMHMM and AllerTop servers. The physicochemical properties suggested that all vaccine constructs are highly efficient, thermostable, non-antigenic, and non-allergenic (**Supplementary Table S8**).

### 3.9 Analysis of 2D and 3D structures

The SOPMA server was employed for 2D structural analysis of the vaccine constructs (V1, V2, V3, and V4). Specifically, V1 consists of 13.15% α-helices, 76.41% random coils, and 10.44% extended strands. V2 includes 31.17% α-helices, 57.30% random coils, and 11.53% extended strands; V3 includes 37.61% α-helices, 51.66% random coils, and 10.73% extended strands; and V4 contains 41.27% α-helices, 53.94% random coils, and 4.79% extended strands. GlobPlot analysis identified two globular regions (3–143 and 438-555) within the V2 vaccine construct, along with seven disordered regions (1-5, 144-267, 290-335, 371-395, 411-437, 455-467, 485-509). V4 has two globular regions (1-172, 467-584) and seven disordered regions (3-8, 173-296, 319-364, 400-424, 440-466, 484-496, 514-538). Reported disordered regions of both V2 and V4 covered the adjuvant, MHC I, MHC II, and segments of B cell epitopes. First, globular regions of V2 and V4 covered the entire adjuvant region, terminating in MHC I epitopes, while the second globular region largely included B cell epitopes. The 3D structures of vaccine constructs were anticipated using the trRosetta server. The structural efficacy of vaccine designs was evaluated using a Ramachandran plot and ERRAT analysis by the SAVES server. The V2 and V4 had ERRAT quality factor values of 94.36% and 98%, suggesting that the structures were of good quality. The Ramachandran plot demonstrated that 90.6% of residues in V2 and 98.6% of residues in V4 were situated inside the preferred regions, verifying the stability of the 3D structure.

### 3.10 Population coverage analysis

**Figure 3:**
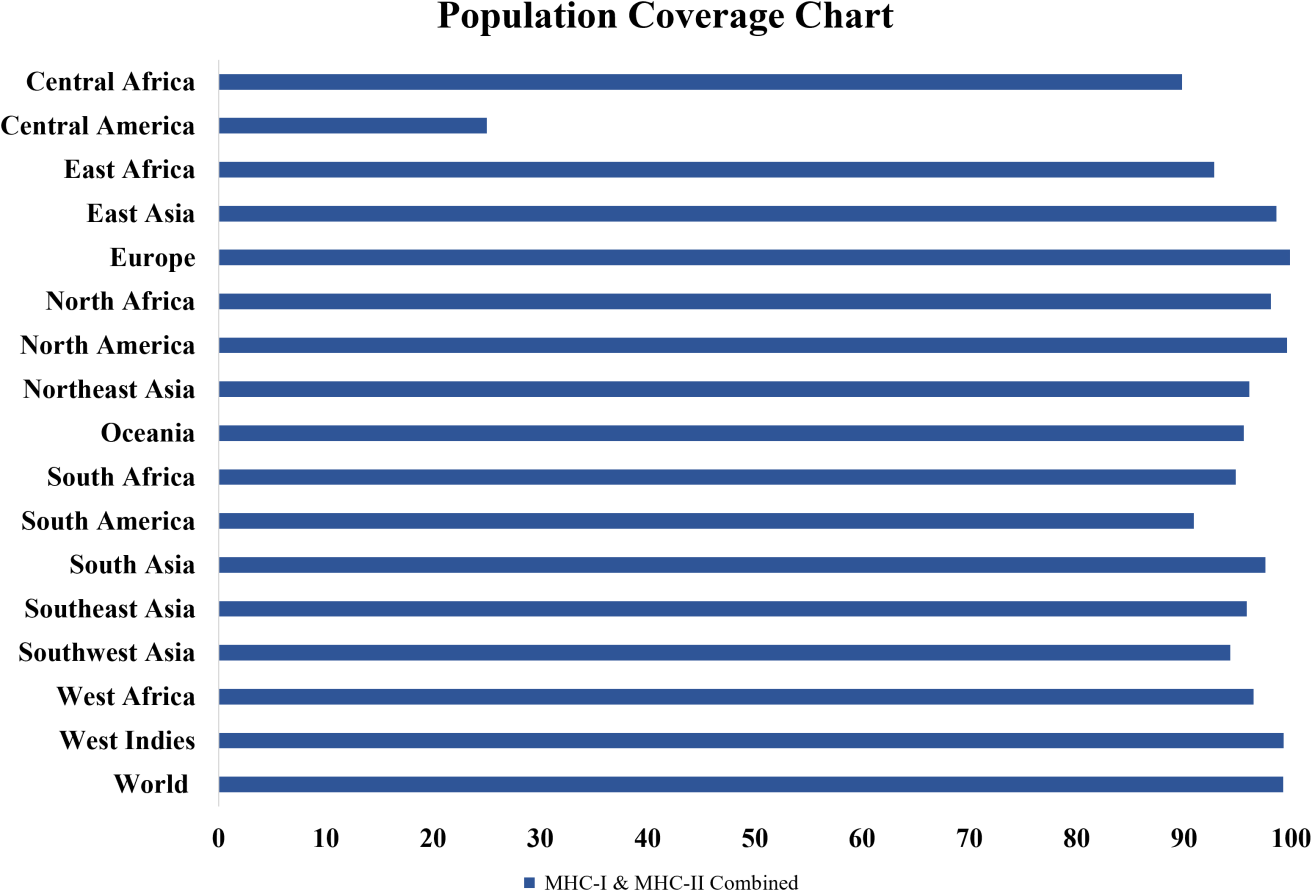
Population coverage analysis of the selected T-cell epitopes’ combined MHC-I and MHC-II alleles. Selected MHC-I and MHC-II epitopes are expected to cover 99.26% of the global population.

The population coverage tool from the IEDB was utilized to estimate the global population coverage of the chosen T-cell epitopes. According to the results, selected MHC-I and MHC-II epitopes are expected to cover 99.26% of the global population (**Figure 3**). This high coverage shows that the vaccine can induce immunological responses in a wide range of populations around the world, confirming its goal effectiveness.

### 3.11 Molecular docking

**Figure 4:**
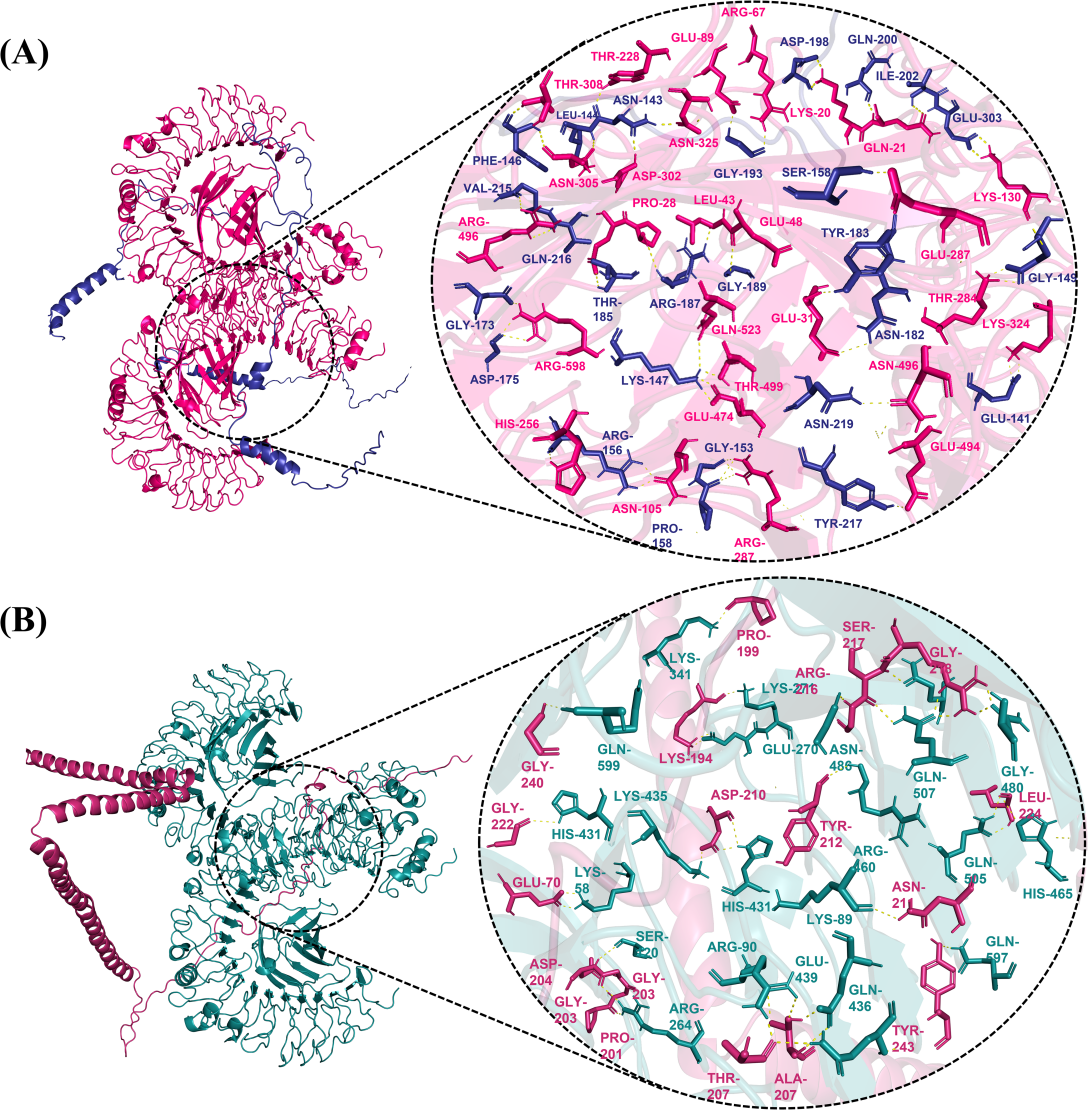
Interaction visualization of the docked complex **(A)** Human TLR4 (Pink) interaction with the V2 vaccine construct (blue), illustrating a vaccine candidate in complex with TLR4. **(B)** The V4 vaccine construct’s (pink) interaction with human TLR4 (teal) demonstrates that the vaccine candidate interacts with TLR4.

Vaccines can interact with immunological receptors, such as Toll-like receptors, to trigger robust immune activities. The ClusPro server was used for molecular docking analysis and vaccine affinity testing using the human immunological receptors TLR4 and 5. The software generated docked models of vaccines and receptors and their binding affinities. PyMOL software was used to visualize the interactions between vaccine constructs (V1, V2, and V4) with receptor TLR4 and V3 with TLR5, providing insights into vaccine interactions and immune response modulation. The docking of V1 with TLR4 resulted in a binding energy of -1493.8 and the formation of 25 hydrogen bonds with TLR4. The V2-TLR4 complex showed a binding affinity of -1434.4, and 40 hydrogen bonds were among them. For V3, the docking score with TLR5 was -1507, with 23 hydrogen bonds observed. In contrast, the V4-TLR4 complex exhibited a docking score of -1434.4 and 35 hydrogen bonds. The V2 and V4 complexes demonstrated the highest docking scores with TLR4, indicating stronger interactions than V1 and V3 (**Figure 4**). Therefore, they were prioritized for further analysis.

### 3.12 Bond length measurement

Bond lengths between interacting residues of TLR4 and vaccines V2 and V4 were measured using Pymol software. The length between all the hydrogen bonds of V2 and TLR4 ranged from 1.7 and 2.9 Å, while in V4-TLR4, bond lengths varied between 1.7 to 2.9 Å. Since all the bond lengths are within the favorable bond length of a typical hydrogen bond, this indicates the stronger binding of our vaccines with the receptor.

### 3.13 NMA evaluation

The iMODS web server was used for structural analysis of selected docked complexes, modifying the force field over various time intervals. Peak areas on the deformability graph represent residues in the primary chain that experience deformation within the receptor-vaccine complexes. These high deformability areas aid in identifying linkers in the main chain (**Supplementary Figures S1A**, **S2A**). Furthermore, **Supplementary Figures S1B** and **S2B** show the B-factor as an indicator of protein mobility. The predicted eigenvalue for the V2-TLR4 complex was 5.986407e-10, while that for V4 was 8.589711e-10, indicating motion stiffness corresponding to each normal (**Supplementary Figure S1C**, **S2C**). The variance bar illustrates individual (purple) and cumulative (green) variances for each complex’s normal mode. A negative correlation was observed between variance and covariance (**Supplementary Figure S1D**, **S2D**). The covariance map illustrates motion between molecules in the complexes, displaying correlated (red), uncorrelated (white), and anticorrelated (blue) atomic motions, which indicate linked movements among residue pairs in **Supplementary Figures S1E**, **S2E**. An elastic network map was also generated, showing a pair of atoms connected by springs, with colored dots depicting stiffness and assembly between atoms of larger molecules and darker gray indicating more rigid springs (**Supplementary Figures S1F**, **S2F**).

### 3.14 MD simulation

The molecular dynamics simulations were performed for 100 nanoseconds using AMBER 21.

#### 3.14.1 RMSD

**Figure 5:**
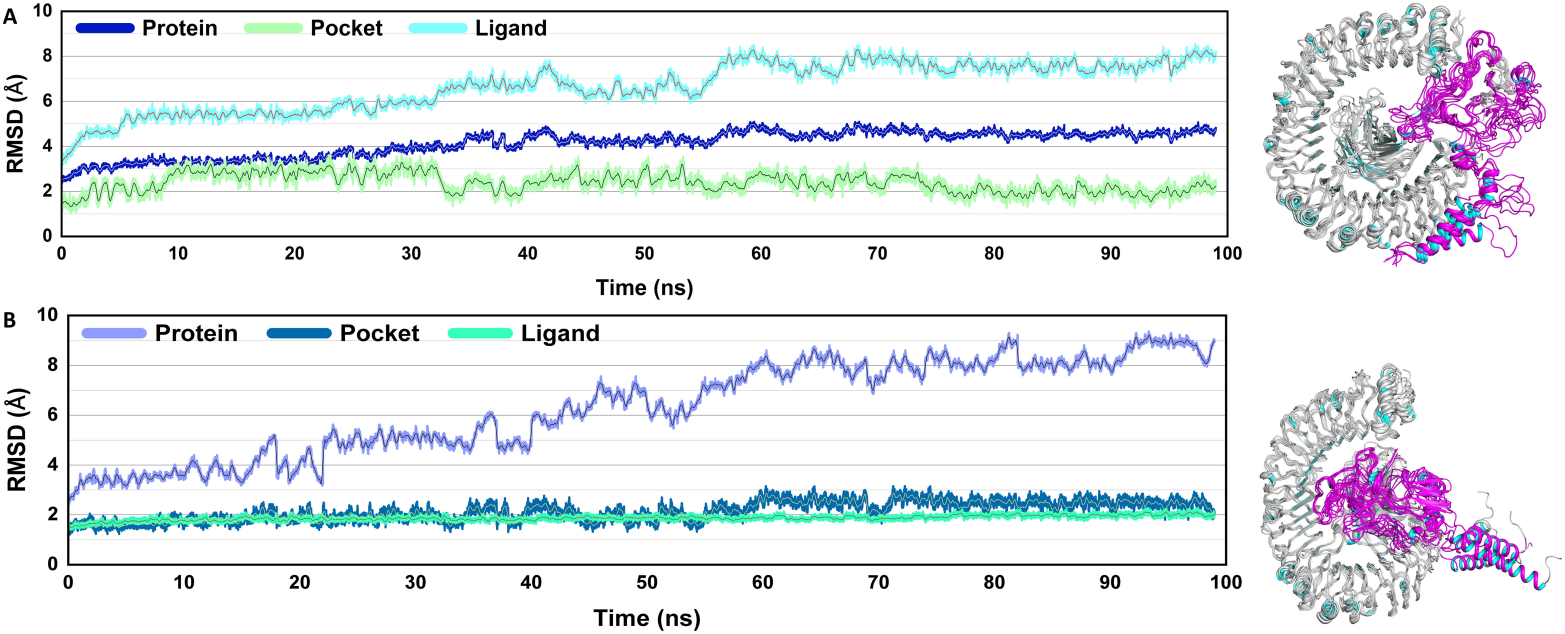
Root mean square deviation (RMSD): The structural integrity of the systems is proven by the RMSD study. The V2 vaccine complex shows the highest variability, while the V4-TLR4 complex shows the most stable behavior with minimal variations. A stable but dynamic interaction is exhibited by the V4-TLR4 complex’s intermediate stability. RMSD graphs of the vaccine docked with TLR4 **(A)**, V2 **(B)** V4.

To determine the dynamic stability of the ligands (V2, V4), protein pocket, and protein, the root mean square deviation (RMSD) was computed over 100 ns using actual geometries of the molecules (**Figure 5**). The analysis was critical for understanding the dynamics of the system and revealing important interactions between vaccines and receptors. The RMSD plot of the complexes revealed that the protein, pocket, and ligands were almost stable throughout the simulation. Only minor fluctuations were observed throughout the simulation period in the case of the V2-TLR4 complex. However, a consistent rise in the RMSD of the V4-TLR4 complex was observed, which reached a maximum value. Overall, decreased volatility suggests the vaccine combinations may be highly stable and immunogenic.

#### 3.14.2 RMSF

**Figure 6:**
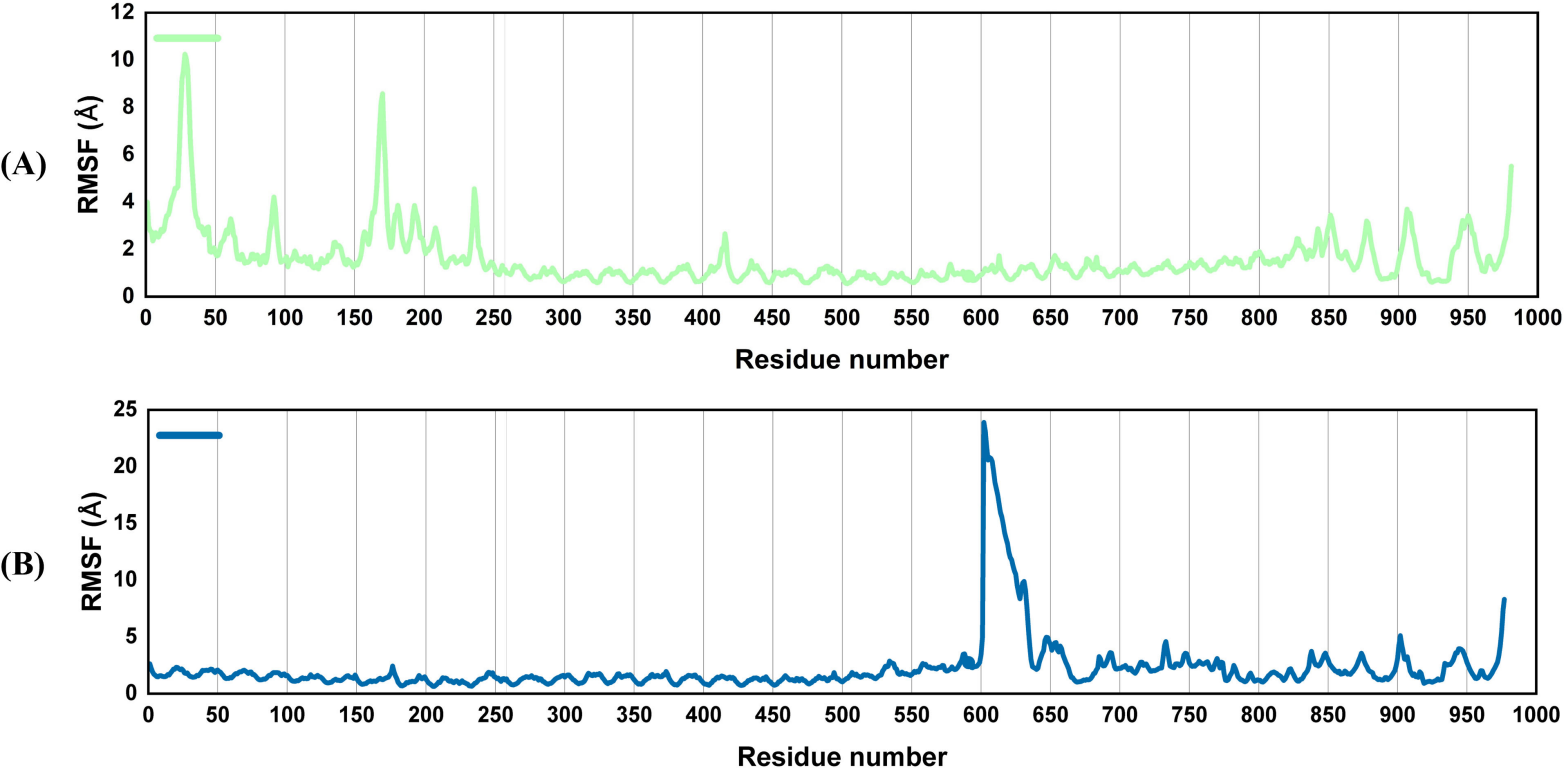
Root mean square fluctuation (RMSF): The output presents flexibility at the level of a residue. Vaccine-TLR4 complex with minimum volatility reveals that the receptor is heavily stabilized in it. The V2-TLR4 system, however, is highly flexible, while the V4-TLR4 complex fluctuates moderately, particularly in dynamic regions such as loops. RMSF graphs of the vaccine docked with TLR4 **(A)**, V2 **(B)** V4.

Root mean square fluctuations (RMSF) explain alterations in protein area observed during the simulation. The flexibility of every residue is thus assessed to gain a better understanding of how vaccines and TLR4 binding affect the flexibility of the complexes. The protein’s amino acid residues remained stable during the simulation, as evidenced by **Figure 6**, which revealed relatively few non-significant changes in the atoms of both complexes. This lends support to the idea that after the interaction of the vaccines with the receptor, the complexes remained stable. The vaccine molecule has a high affinity for receptors and forms stable complexes (**Figure 6**). Since some residues in the complexes, i.e., the residues at the C and N-terminal in the V2-TLR4 complex and 600–650 residues in the V4-TLR4 complex, have intrinsically flexible regions, their RMSF values vary significantly. This is similar to the loop portions, adjuvants, and linker sequences.

#### 3.14.3 Radius of gyration

**Figure 7:**
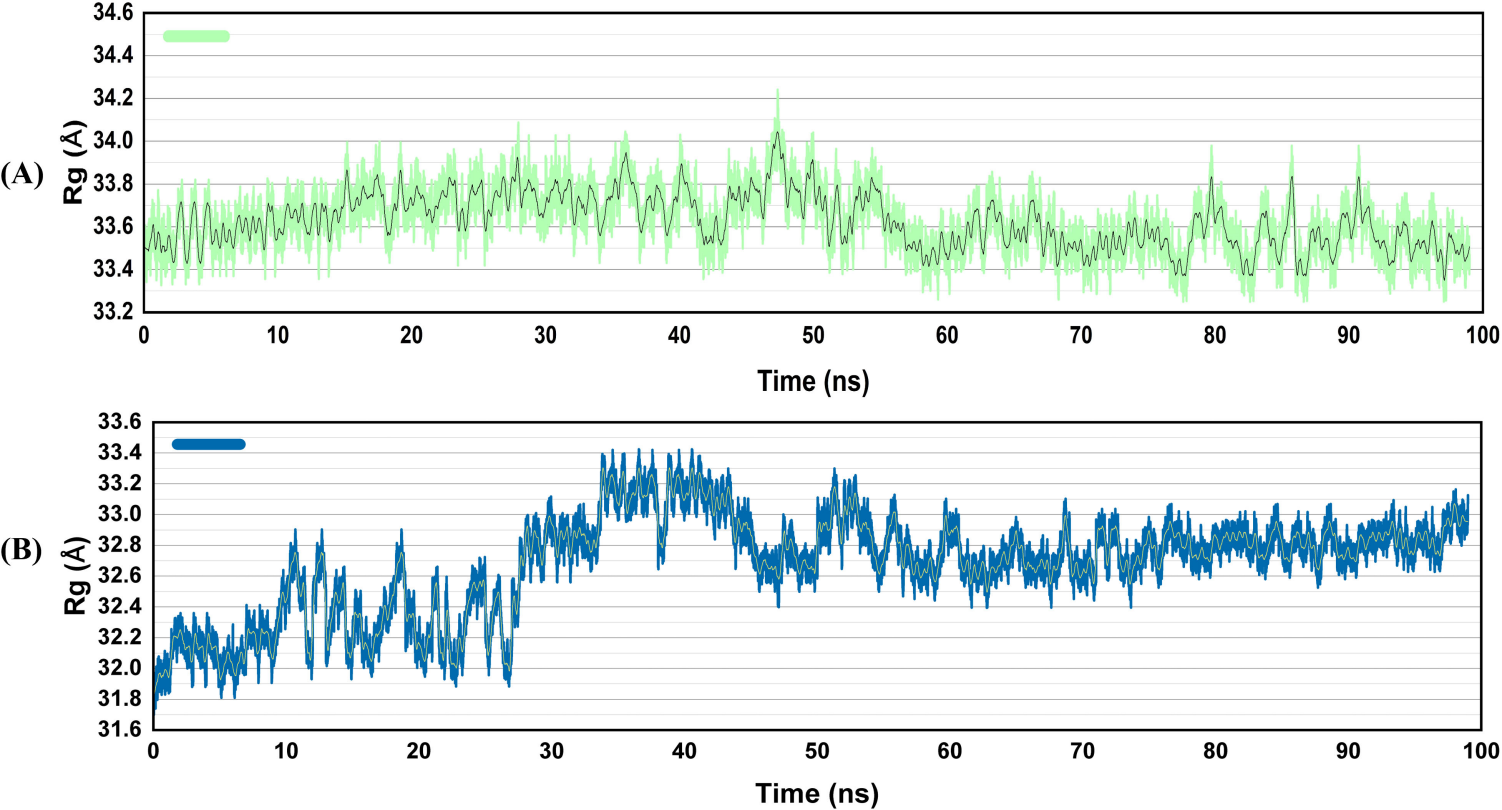
Radius of gyration of the vaccines docked with TLR4 **(A)**, V2 **(B)** V4. Structural compactness is expressed by the analysis of Rg. A compact and stable structure is demonstrated by the V2-TLR4 complex’s preservation of the largest and most consistent Rg. Although the V4-TLR4 complex is of intermediate tightness with pronounced dynamic behavior, the vaccine’s system retains the smallest and most fluctuating Rg, as it is flexible.

The radius of gyration (Rg) of the docked complexes was also determined. It quantifies Ca atomic displacement from the center of mass, whereas protein folding stability examines a level of compactness across the simulation time. The V2-TLR4 complex showed almost stable Rg values. However, in the case of the V4-TLR4 complexes, the values remained stable till 30 ns. After that, a rise in Rg values was observed around 33.4 ± 0.2 Å (**Figure 7**). Then, a slight decrease was observed between 40 to 50 ns, and the Rg then came to equilibrium. These tiny oscillations may allow for minor alterations in the binding interface, perhaps enhancing the TLR’s capability to recognize diverse epitopes.

#### 3.14.4 SASA

**Figure 8:**
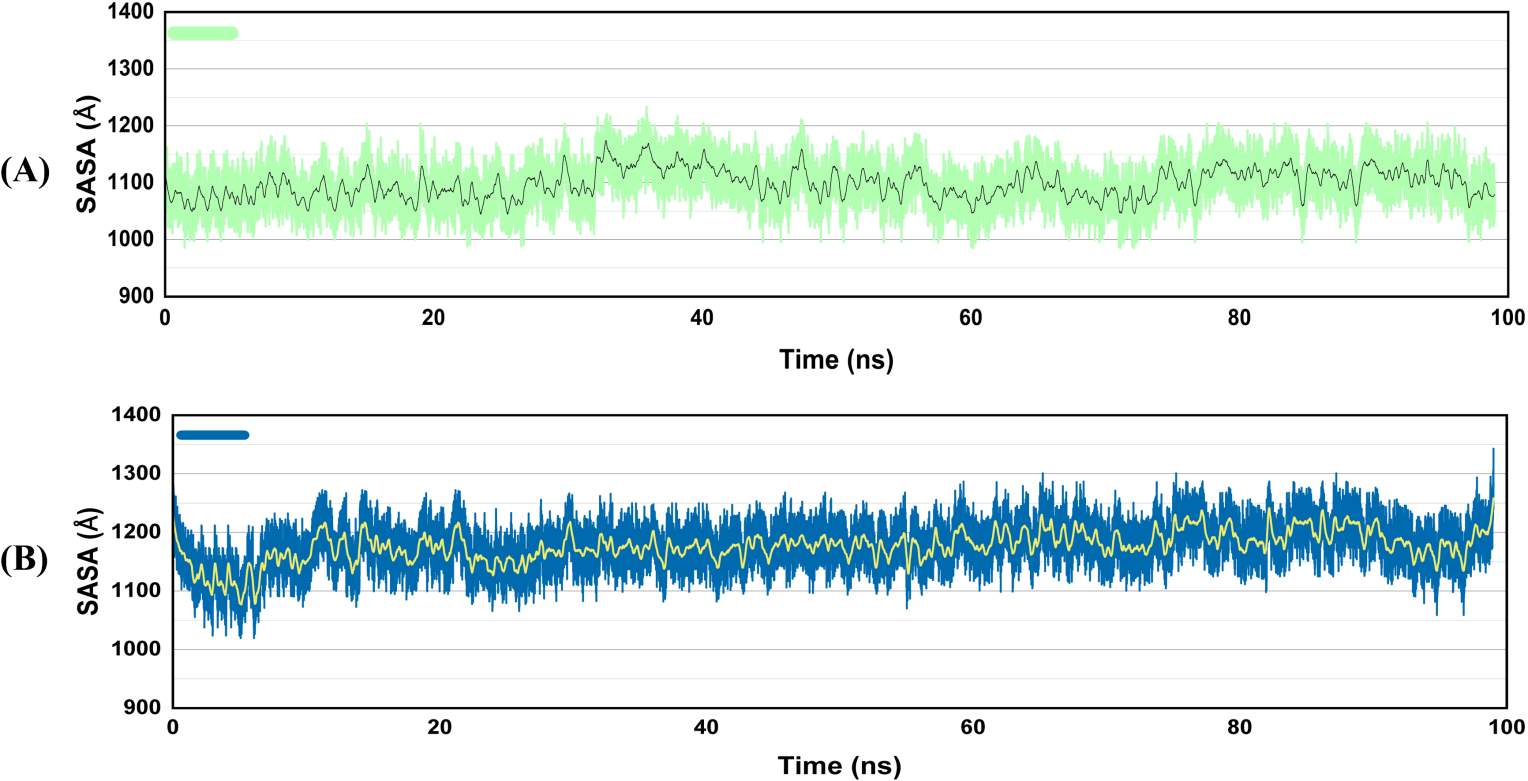
SASA graphs of the vaccine docked with TLR4 **(A)**, V2 **(B)** V4. The enhanced SASA of the complexes suggested that the V2 and V4 vaccine constructs undergo a structural change when they bind to TLR4, quite possibly by presenting a larger surface to the solvent surrounding them.

Solvent accessible surface area, or SASA, is the quantity of surface area that solvent molecules can reach on a biological molecule (such as a protein) or another molecular framework. It is the extent to which the solvent can come into contact with the atoms on the surface of a protein. Both vaccine-TLR4 complexes were stable throughout the simulation period, as seen by the SASA graph (**Figure 8**). The stability suggests that the vaccines bind to TLR4 securely, burying significant residues in the interface and possibly enhancing binding stability and immune response elicitation.

### 3.15 Binding free energy calculations

**Figure 9:**
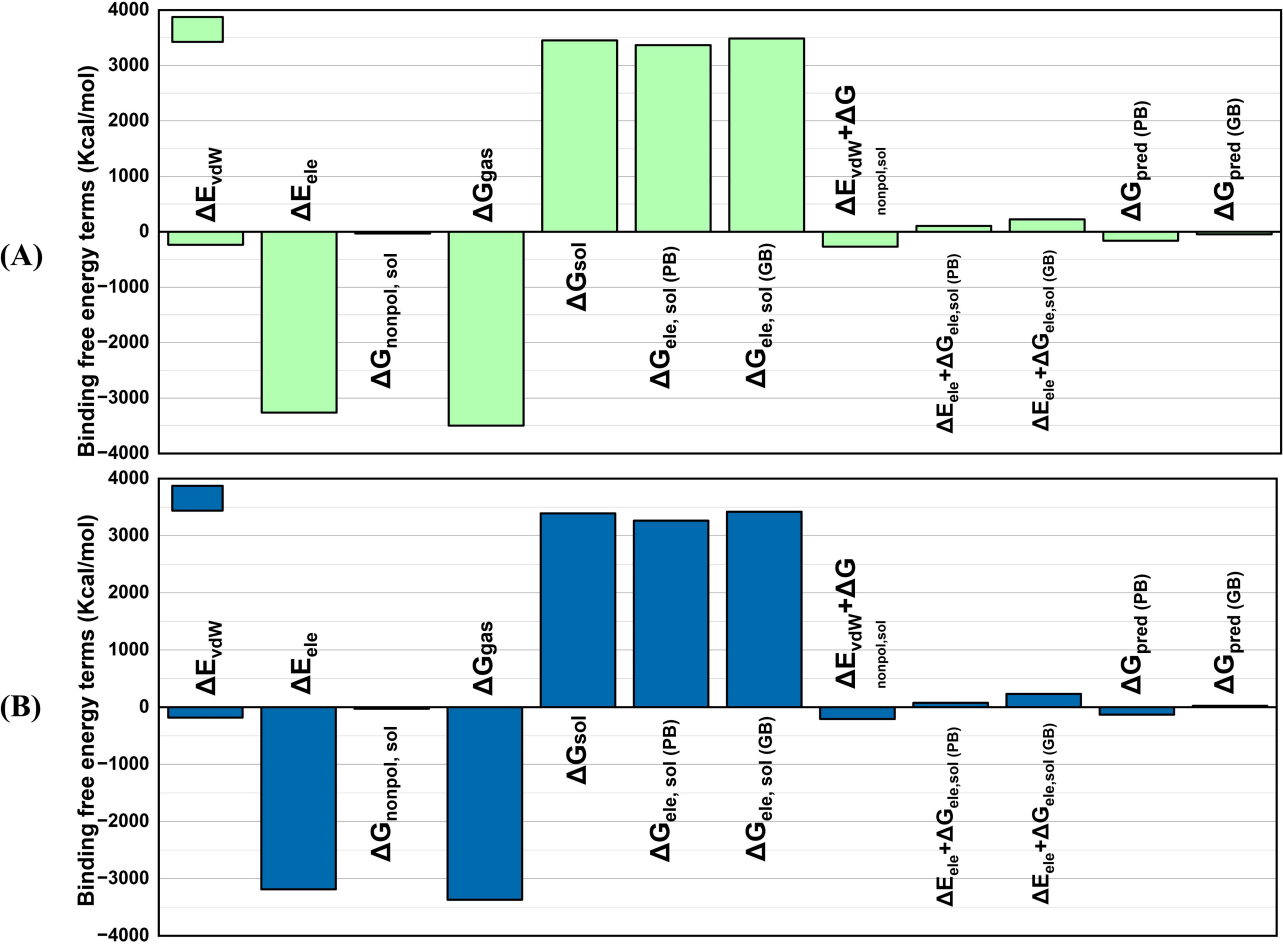
MMPBSA graphs of the vaccine docked with TLR4 **(A)** V2 **(B)** V4. V2-TLR4 has strong binding interactions, with total binding free energies ΔG_pred(PB)_ and ΔG_pred(GB)_ of -161.7315 and -45.09-144.8623 kcal/mol, respectively. Likewise, the V4-TLR4 complex showed these binding free energy values as -132.0631 and 23.1254 kcal/mol, respectively.

The MMPBSA technique is well-known for estimating complex binding free energies with great precision. MMPBSA computes net binding free energy, where negative values indicate high receptor-ligand affinity and positive values indicate low docked stability (**Figure 9**). Determining BFE is vital because it provides information about the stability as well as the strength of molecular interactions, which is required to completely comprehend protein-ligand binding dynamics. The BFE analysis of the vaccine-receptor docked complexes showed a van der Waals (ΔE_vdW_) energy of -235.017 kcal/mol in V2 and -181.8155 kcal/mol in V4 complex. The electrostatic energy (ΔE_ele_) of the V2 complex was -3262.6418 kcal/mol, while that of the V4 showed -3187.0594 kcal/mol. The solvation energy (ΔG_sol_) and non-polar solvation energy (ΔG_nonpol, sol_) of the V2-TLR4 complex were calculated as 3452.5688 and -32.6564 kcal/mol, respectively, while that of the V4 complex was 3392.0003 and -26.6135 kcal/mol. V2 and TLR4 have strong binding interactions, with total binding free energies ΔG_pred (PB)_ and ΔG_pred(GB)_ of -161.7315 and -45.09-144.8623 kcal/mol, respectively. Similarly, the V4-TLR4 complex showed these binding free energy values as -132.0631 and 23.1254 kcal/mol, respectively. This contributes to an effective immune response. The free energy change in the gas phase (ΔG gas) of V2 and V4 complexes was -3497.6588 and -3368.8749 kcal/mol, respectively. The lower ΔG gas indicates that the gas phase contributes more to the binding affinity than the solvation effect, indicating a positive relationship between the molecules.

### 3.16 PCA analysis and free energy landscape

**Figure 10:**
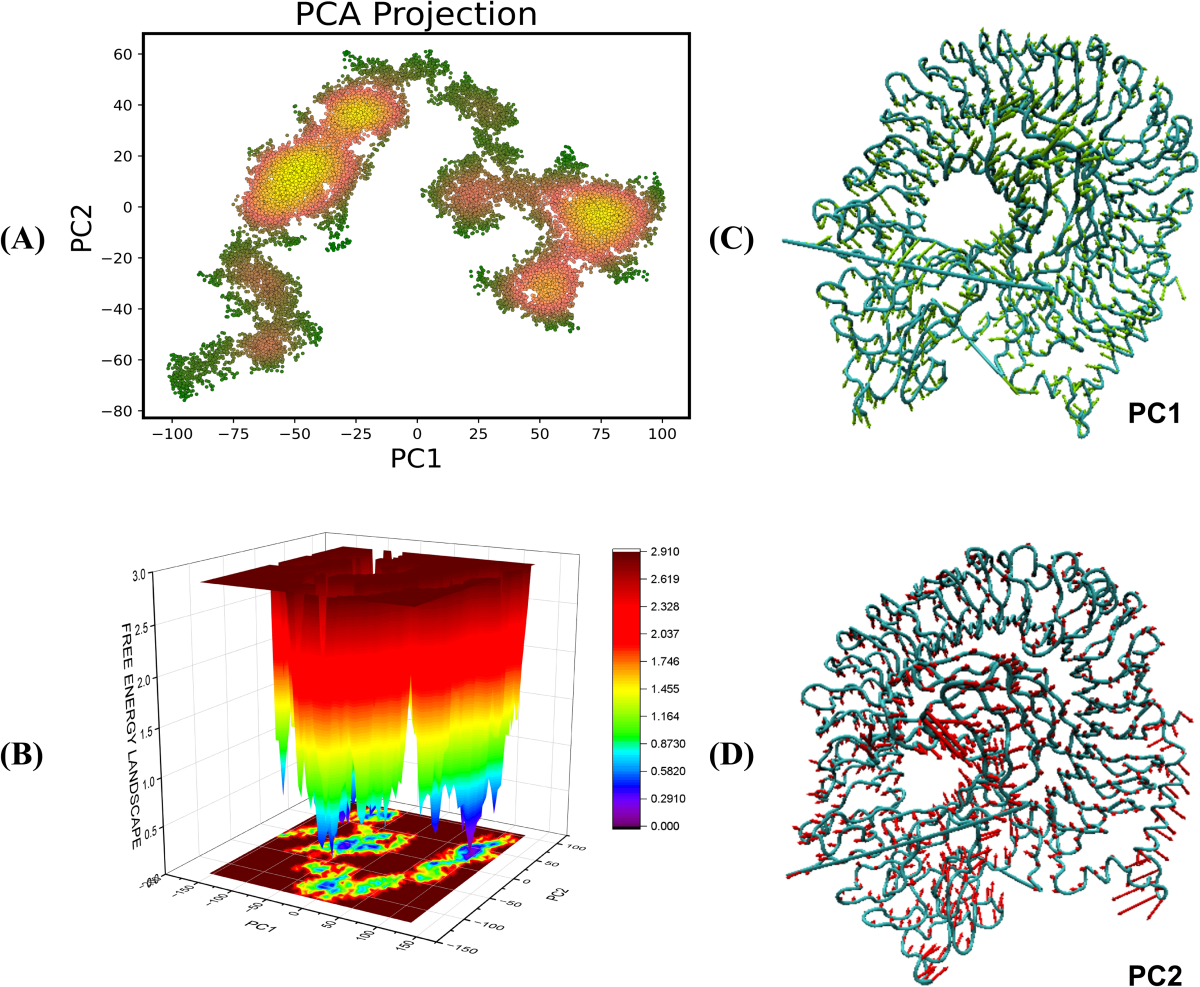
Principal component analysis and free energy landscape projection of the V2-TLR4 complex. **(A)** The PCA plot color gradient from green to yellow/red represents the simulation’s time progression, with green indicating the early phase and yellow/red indicating later stages. **(B)** FEL plot represented in the form of ΔG° values in the FEL plots **(C, D)** representing the PC1 and PC2 components for the analysis.

**Figure 11:**
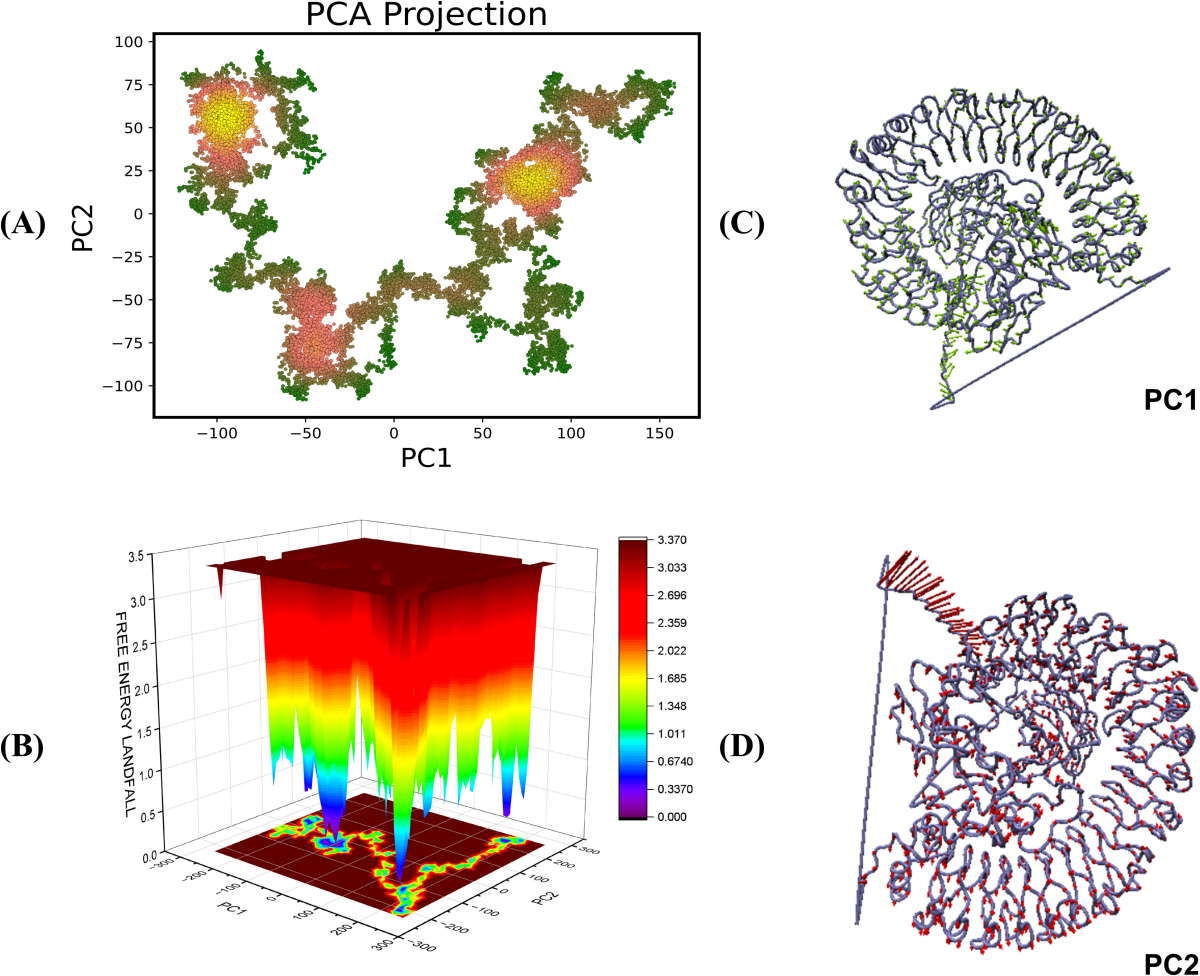
Principal component analysis and free energy landscape projection of the V4-TLR4 complex. **(A)** The PCA plot color gradient from green to yellow/red represents the simulation’s time progression, with green indicating the early phase and yellow/red indicating later stages. **(B)** FEL plot represented in the form of ΔG° values in the FEL plots **(C, D)** representing the PC1 and PC2 components for the analysis.

The Principal Component Analysis (PCA) yields vital insights into the conformational dynamics of TLR4-vaccine complexes during molecular dynamics (MD) simulations. PCA reduces motion complexity, allowing us to identify the most common modes along the primary axes, PC1 and PC2, which represent linear combinations of atomic displacements. The color gradient on the PCA plot from green to yellow/red shows the simulation’s time progression, with green representing the initial stage and yellow/red representing the later stages (**Figures 10A**, **11A**). The transition from green to yellow/red implies that the complex is approaching a stable binding state, in which the interaction turns more energetically beneficial. Compact clusters of yellow/red markings show conformations that have been repeatedly sampled and represent stable interaction conformations within the complex. The early green phase is most likely an exploration phase in which the complex finds a suitable binding orientation, but the compact yellow cluster suggests that the complex has reached a stable form, which may be required for effective immune detection. The free energy landscape (FEL), obtained from PC1 and PC2, sheds light on these conformational changes. Warmer hues (red/orange) indicate higher energy, less stable states, whereas cooler hues (yellow/green/blue) indicate lower energy, more stable states (**Figures 10B**, **11B**). This smooth transition from high to low energy shows the complex’s progression to a stable conformation, most probable within the TLR4 interaction pocket. The red areas of the FEL represent less stable conformations, while the blue regions represent most stable conformations.

The PCA and FEL studies show that both the vaccine-receptor complexes undergo initial conformational adjustments to find an appropriate binding orientation before reaching a stable, energetically beneficial binding position. This stability may be crucial for generating the desired immune response, increasing the vaccine’s effectiveness in triggering TLR-mediated signaling. Furthermore, the conformations of the complexes are visualized in **Figures 10C, D** and **11C, D**.

### 3.17 DCCM

**Figure 12:**
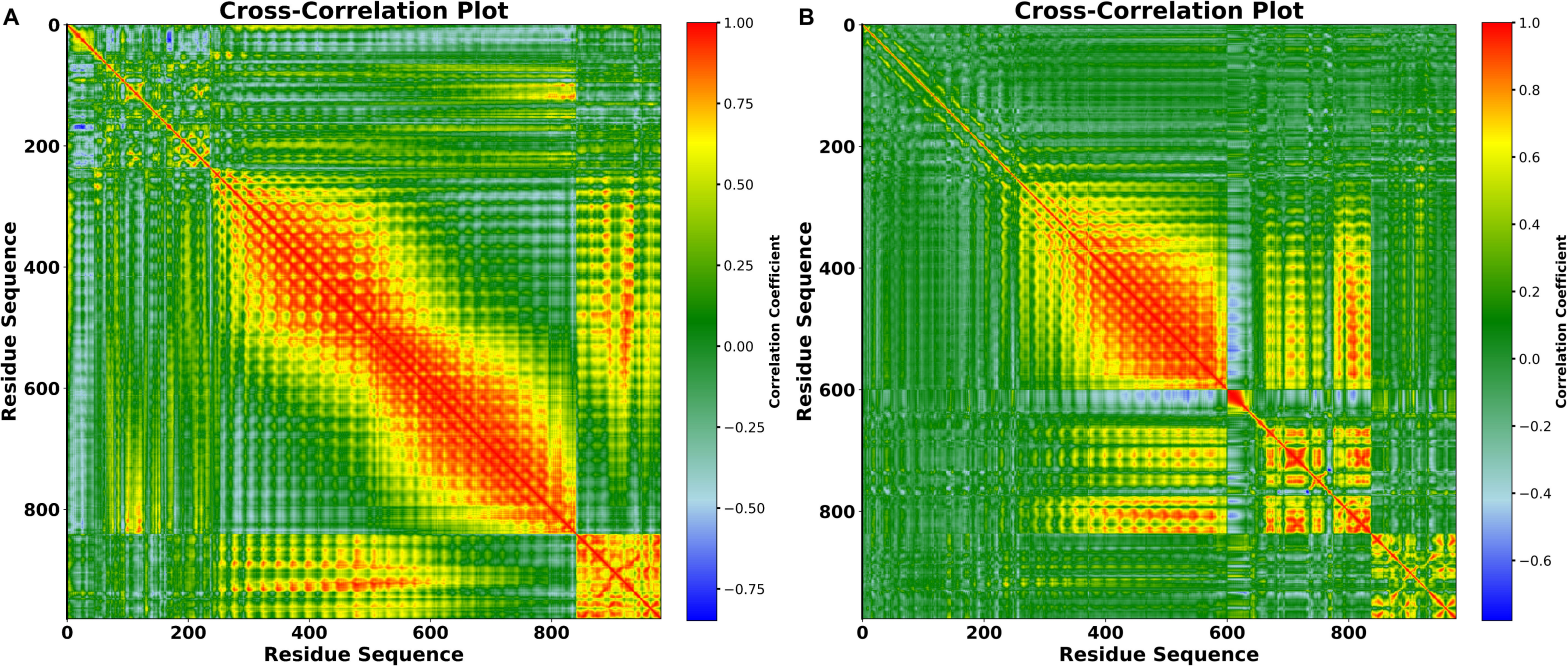
Dynamic Cross-Correlation Map of the vaccine docked with TLR4 **(A)** V2 **(B)** V4. Positive, and negative movements between paired residues in the two chains are indicated by the colors red, and blue in the DCCM figure, respectively.

The collective rotation of Ca backbone atoms in a protein’s global domain influences the change of protein conformations from one functional state to the next. To analyze these displacement patterns, we developed and studied a dynamic cross-correlation matrix (DCCM) (**Figure 12**). It illustrates the collective fluctuation of Ca atoms and uses relative motion measurements to reveal the interactions between vaccines and TLR4. Positive correlations indicate that the vaccines and TLR4 in a complex move in the same direction (parallel direction), whereas negative correlations indicate that the vaccines and receptor move in the opposite direction. The color red represents a positive correlation; green and yellow show slight correlations and local displacement; and blue denotes a negative correlation. The depth of the hue represents a stronger positive or negative correlation, showing the degree of relationship. Huge red blocks in the diagonal and off-diagonal regions show that distinct domains or components of the vaccine-TLR4 complex move in a highly coordinated manner. Particularly, regions around residues 200-800 in the V2 complex, while 300 to 600 in the V4 complex, have significant positive correlations, showing that these areas move together during the simulation, potentially indicating functional or physically interconnected domains. Smaller green and yellow areas, on the other hand, show places with mild residue correlation, indicating potential sites of mechanical coupling or functional antagonism.

### 3.18 Analysis of post-translational modifications

**Figure 13:**
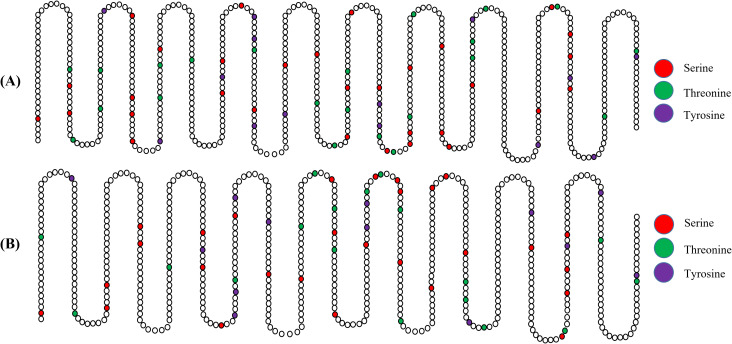
Post-translation modification sites analysis of vaccine constructs predicted phosphorylation and N-glycosylation sites in V2 **(A)** and V4 **(B)**.

Phosphorylation and N-glycosylation sites, important for the folding and functionality of the proteins, were predicted for the final vaccine constructs to demonstrate their ability to undergo these modifications (**Figure 13**). Phosphorylation site prediction for the V2 construct yielded 56 sites (12 Tyr, 27 Ser, and 17 Thr residues), while the V4 construct contained 67 phosphorylation sites (14 Tyr, 31 Ser, and 22 Thr residues. Analysis of glycosylation sites revealed one N-glycosylation site in V4 at position 438 (NKTD) and two N-glycosylation sites in V2 at positions 13 (NLTV) and 409 (NKTD).

### 3.19 Conformational B cell epitopes

The Ellipro server identified vaccine residues involved in conformational B-cell epitope formation. Seventeen B-cell epitopes were identified in V2, with scores in the range of 0.551 to 0.967, consisting of 3–35 residues. For V4, fourteen conformational epitopes were identified, with scores in the range of 0.38 to 0.95 and comprising 3–75 residues (**Supplementary Tables S9** and **S10**).

### 3.20 Immune simulation

The C-ImmSim web server was employed for testing the immunogenic profile of V2 and V4. Immunogenicity increased after three vaccine doses, leading to increased activation of B cells, T_C_ cells, NK cells, and T_h_ cells. This demonstrated improved immune response dynamics with each subsequent injection. **Supplementary Figures S3A** and **S4A** depict the primary antibody responses, showing a significant increase in IgM and IgG following the third vaccine dose. Graphs in **Supplementary Figures S3B** and **S4B** show a considerable increase in helper T-cell production, indicating positive immunological results. Graphs in **Supplementary Figures S3C** and **S4C** demonstrate that the immune response remained vigorous and consistently high after the third injection. Graphs in **Supplementary Figures S3D** and **S4D** indicate there is an apparent rise in the population of TC cells as memory grows.

### 3.21 Codon optimization and in silico cloning

The codon adaptation of V2 and V4 was conducted through the Jcat tool. The CAI value for V2 was 0.98, indicating high expression potential, with a GC content of 50.150%. Similarly, the CAI value for V4 was 0.96, with a GC content of 51.65%, respectively. After codon adaptation, V2 and V4 vaccines were inserted into pET-28a (+) for cloning, and their final clone lengths were 7034 bp and 7121 bp, respectively (**Supplementary Figure S5**).

## 4 Discussion

*Yersinia enterocolitica* is a Gram-negative bacterium that causes *yersiniosis* in humans (1). It is primarily spread by contaminated food or water and causes enterocolitis with symptoms such as diarrhea, stomach discomfort, and fever, which frequently mimics appendicitis in youngsters (7). It is resistant to beta-lactam antibiotics due to beta-lactamase activity and fluoroquinolones through gyrA mutations or efflux mechanisms, underlining the need for effective vaccinations and resistance management.

*Y. enterocolitica* is a rapidly emerging enteric pathogen with clinical manifestations ranging from self-limited enterocolitis to potentially fatal systemic infections and post-infection sequelae, which include reactive arthritis and erythema nodosum (15, 70). It is not quite yet a manageable-for-vaccine infection, recent reports highlight its growing significance. It has been identified as among the top zoonotic organisms of concern by the European Food Safety Authority (EFSA), which emphasizes its importance in foodborne and zoonotic transmission (https://www.efsa.europa.eu/en/efsajournal/pub/9106). This emphasizes the need for preventive strategies against high-risk populations and occupationally exposed individuals. Furthermore, *Y. enterocolitica* has experienced a global rise in antimicrobial resistance (AMR), especially against third-generation cephalosporins and other critical antibiotics, which is a major public health issue. The potential for the emergence of multi-resistant strains could severely limit treatment options and lead to more complicated disease outcomes (70). As *Y. enterocolitica* is present in animal reservoirs such as rodents, dogs, pigs, and environmental sources, there is a continuing risk of zoonotic transmission in the exposed work populations, especially within the commercial meat, poultry, and seafood production, as well as food processing areas. Surveillance reports from organizations such as the CDC and WHO emphasize the evolving risk posed by AMR in zoonotic pathogens (https://www.cdc.gov/yersinia/about/index.html), (https://www.who.int/publications/i/item/9789241509763). Due to these emerging risks, there could be a dual purpose in developing a targeted vaccine strategy that would decrease clinical burden in high-risk groups and prevent zoonotic transmission. A vaccine based on this design would be consistent with the One Health approach to addressing emerging infectious threats, proactively (71). By focusing on *Y. enterocolitica*, we aim to reduce the burden on healthcare systems, mitigate the risks of AMR, and enhance public health preparedness in the face of evolving challenges.

This study aimed to identify novel therapeutic targets through the analysis of *Y. enterocolitica* core proteome. Multiple web servers were utilized for selecting potential vaccine candidates against *Y. enterocolitica* that were highly conserved in the majority of pathotypes, making the findings applicable to numerous strains of pathogens. Bacterial membrane proteins are essential for membrane integrity maintenance, molecular transport, and pathogenicity, as they are the initial molecules to interface with host cells. These proteins are promising candidates for vaccine design (72). Since the primary target groups of *Y. enterocolitica* are immunocompromised individuals, and its incidence rate is higher in infants and young children, although all ages are at risk (73). Therefore, the vaccine designed in this study or any vaccine designed against enteric yersiniosis should provide immunity to these target groups or the vulnerable population.

Porins are crucial outer membrane proteins in bacteria that play an important role in physiology and pathophysiology. The porin protein (WP_019079224.1) helps transport nutrients and tiny hydrophilic compounds across the bacterial outer membrane. Porins are a promising target for vaccine design due to their surface accessibility and immunogenic potential (74, 75). Furthermore, TonB-dependent siderophore receptors (WP_050161901.1) are essential for the survival and virulence of *Y. enterocolitica*. They allow for iron uptake in host settings with limited iron availability (76). Their surface exposure, wide distribution, protective immunogenicity, and essential roles in pathogenicity make them excellent candidates for vaccine development (77). These two vaccine candidates were used for the identification of epitopes and vaccine design. The capsular antigen F1, the type 3 secretion system (T3SS) component, and the effector low-calcium response V antigen (LcrV) are currently in clinical trials and show promise in rodent models (78). However, the protection level varies among different non-human primate species, and there is a weak correlation between antibody titers and protection (79). Additionally, antibody levels produced by these vaccines in humans vary significantly, and elicit a limited T cell response (80). Recent research, including our findings, has suggested that cell-mediated immune responses are crucial in offering protection against *Yersinia* infections, particularly pneumonic plague; therefore, subunit vaccines are unlikely to provide robust efficacy since they mainly result in an antibody-driven immune response (81). Unlike traditional agents (F1, T3SS, & LcrV) which may produce strong humoral but weak cellular responses, our selected proteins (WP_019079224.1 and WP_050161901.1) were designed to stimulate both B and T cell responses.

Vaccine designing with mapped epitopes is an innovative method for inducing a targeted immune response against infectious agents. Selected MHC-I, MHC-II, and B-cell epitopes from the two vaccine targets were joined using the EAAAK, GPGPG, AAY, and KK linkers, which ensure effective epitope separation. First, EAAAK was used for stiffness to enhance bifunctional catalytic activity and fusion protein stability (82). The GPGPG linker was utilized to improve the ability to break junctional immunogenicity and to generate a T cell immune response, resulting in the restoration of immunogenicity for individual epitopes (83). Then, AAY and KK linkers were used, respectively. The AAY linker is utilized to connect protein domains, which improves immune cell access to antigenic regions. It also projects the structural integrity of vaccine antigens. This keeps them stable and ensures effective immunogenicity (84). The KK linker is commonly employed to bind a protein antigen to a carrier protein. Its positive charge enhances the contact between the antigen and the carrier, which boosts the immunological response (85). Four adjuvants, including HBHA-conserved, L7/L12 ribosomal, flagellin, and β-defensin, were employed to enhance the immunogenicity of the designed constructs (86). The secondary and tertiary structures of the prioritized vaccines were developed and verified. Understanding the complex formation and interaction between vaccines and host immune receptors is important to measure vaccine effectiveness. Previous studies have revealed the importance of human TLR receptors in detecting infectious pathogen peptides and generating immune responses to them (87). TLR4 is a member of the pattern recognition receptor family. It is important in the human immune system as it recognizes infectious pathogen peptides and initiates strong immune responses with high sensitivity and specificity. It is crucial for recognizing and responding to invasive conditions (88). Furthermore, TLR5 activation induces a high antibody titer and combined T_H1_ and T_H2_ cellular immune responses (89). Flagellin, a well-known immunomodulatory adjuvant, binds specifically to TLR5, stimulating innate immune responses by activating pro-inflammatory signaling pathways. This interaction improves antigen presentation, activates adaptive immunity, and facilitates a robust immune response (90). Only the vaccine construct with flagellin (V3) was docked with TLR5 in this study to test the immune potential. Therefore, these two receptors were used for the docking of the vaccine and receptor where V2 and V4 displayed the highest interaction with the least amount of binding energy. The normal mode analysis, binding free energy calculation, PCA analysis, molecular dynamic simulations, and DCCM analysis were also performed to ensure the stability of the best complexes. These analysis results predicted strong and efficient binding of the V2 & V4 with the immune receptors, providing insights into their significance. As provided by the NMA analysis, lower eigenvalues revealed that the V2-TLR4 and V4-TLR4 complexes required less energy to distort, indicating greater stability. Deformability, variance, covariance, B-factor, and elastic network analysis revealed high interactions between vaccine constructs and TLR4 receptor, implying that they have the potential to elicit robust innate immune responses. Furthermore, the immune simulation results showed strong immune-simulating responses, with repeated antigen exposure increasing T-helper and T-cytotoxic cells, as well as IgG, IgM, IFN-gamma, and IL-2 levels. These results suggest the induction of a significant cellular and humoral immune response after the vaccine administration (91). *In silico* cloning confirmed that designed vaccine constructs could attain high expression levels in *E. coli*. The findings of this work give a solid platform for developing therapeutics against the virus; nevertheless, experimental confirmation is still required to transform these *in silico* predictions into real therapeutic options.

A previous study by Qurat ul Ain et al. (92) integrated subtractive proteomics and immunoinformatics to reveal B-cell-derived T-cell epitopes against *Y. enterocolitica*. Our work builds on and extends that foundation in several key ways. Firstly, a large-scale genome extraction technique was used in subtractive proteomics by formulating a core genome from all the 22 available complete genomes of *Y. enterocolitica*. However, the previous study analyzed epitopes from just nine strains, focusing solely on OmpC isoforms and T cell epitopes. Second, we explicitly targeted both pathogenic and multidrug−resistant (MDR) strains, filling a gap left by Qurat ul Ain and colleagues, who did not address drug resistance. Furthermore, the epitopes used for constructing the vaccine are also novel and extensive *in silico* validation, such as molecular docking followed by 100ns MD Simulations, PCA, DCCM, and binding free energy calculation, are performed in our study to validate our vaccine interaction with immune receptors. Together, these enhancements demonstrate the novelty and rigor of our therapeutic target and vaccine design pipeline.

## 5 Conclusion

In this study, computational approaches were used to design a multi-epitope vaccine for *Y. enterocolitica*. The approach centered on identifying conserved and antigenic proteins in the core proteome and predicting B and T cell epitopes that might stimulate both humoral and cellular immune responses. The vaccine constructs bind strongly to immune cell receptors such as TLR4, indicating their ability to induce robust innate and adaptive immune activation against the pathogen. However, regardless of encouraging *in silico* immunogenicity, experimental validation is required to ensure the vaccine’s efficacy and safety. More *in vitro* and *in vivo* investigations are needed to improve this vaccine and determine its practical application against *Y. enterocolitica* infection.

## Data Availability

The original contributions presented in the study are included in the article/**Supplementary Material**. Further inquiries can be directed to the corresponding authors.

## Supplementary material

Supplementary Figure 1 Normal mode analysis of the V2-TLR4 docked complex **(A)** Deformability **(B)** B-factor **(C)** Eigenvalue- 5.986407e-10 **(D)** Variance **(E)** Covariance **(F)** Elastic Network.

Supplementary Figure 2 Normal mode analysis of the V4-TLR4 docked complex **(A)** Deformability **(B)** B-factor **(C)** Eigenvalue- 8.589711e-10 **(D)** Variance **(E)** Covariance **(F)** Elastic Network.

Supplementary Figure 3 In-silico prediction of immune simulation via the C-ImmSim server for the V2 vaccine construct. **(A)** IgG, IgM, IgG1, IgG2 Antibodies production **(B)** T helper cell population per state **(C)** B cell population per state **(D)** T cytotoxic cell population per state **(E)** Natural killer cell production **(F)** Dendritic cell population

Supplementary Figure 4 In-silico prediction of immune simulation via the C-ImmSim server for the V4 vaccine construct. **(A)** IgG, IgM, IgG1, IgG2 Antibodies production **(B)** T helper cell population per state **(C)** B cell population per state **(D)** T cytotoxic cell population per state **(E)** Natural killer cell production **(F)** Dendritic cell population

Supplementary Figure 5 In-silico cloning of the V2 **(A)** and V4 **(B)** into the E. coli pET28a vector. The vector, pET-28(+), was 5369 bp in length before the insertion of the chimeric vaccines, V2 and V4. **(A)** The total clone length of the V2 vaccine after insertion into the plasmid was 7034 bp. **(B)** The total clone length of the V4 vaccine after insertion into the plasmid was 7121 bp.
