# Supplementary figures and images for: Screening of novel therapeutic targets and chimeric vaccine construction against antibiotic-resistant Yersinia Enterocolitica
Source: Front Immunol. 2025 Jul 4;16:1555248. doi: 10.3389/fimmu.2025.1555248 (PMC12271202; doi:10.3389/fimmu.2025.1555248)

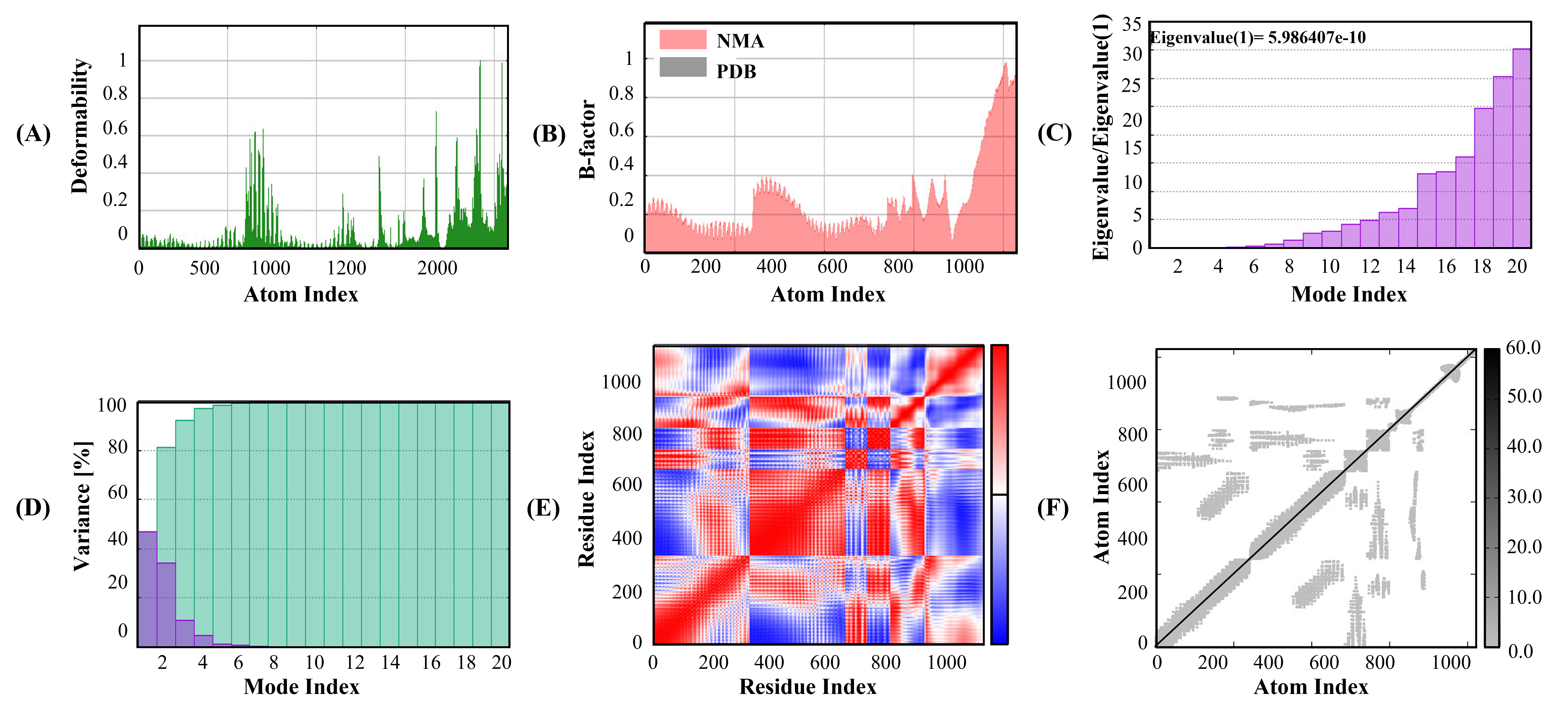

Supplement: Supplementary Figure 1 — Normal mode analysis of the V2-TLR4 docked complex (A) Deformability (B) B-factor (C) Eigenvalue- 5.986407e-10 (D) Variance (E) Covariance (F) Elastic Network. [file Image1.tif]

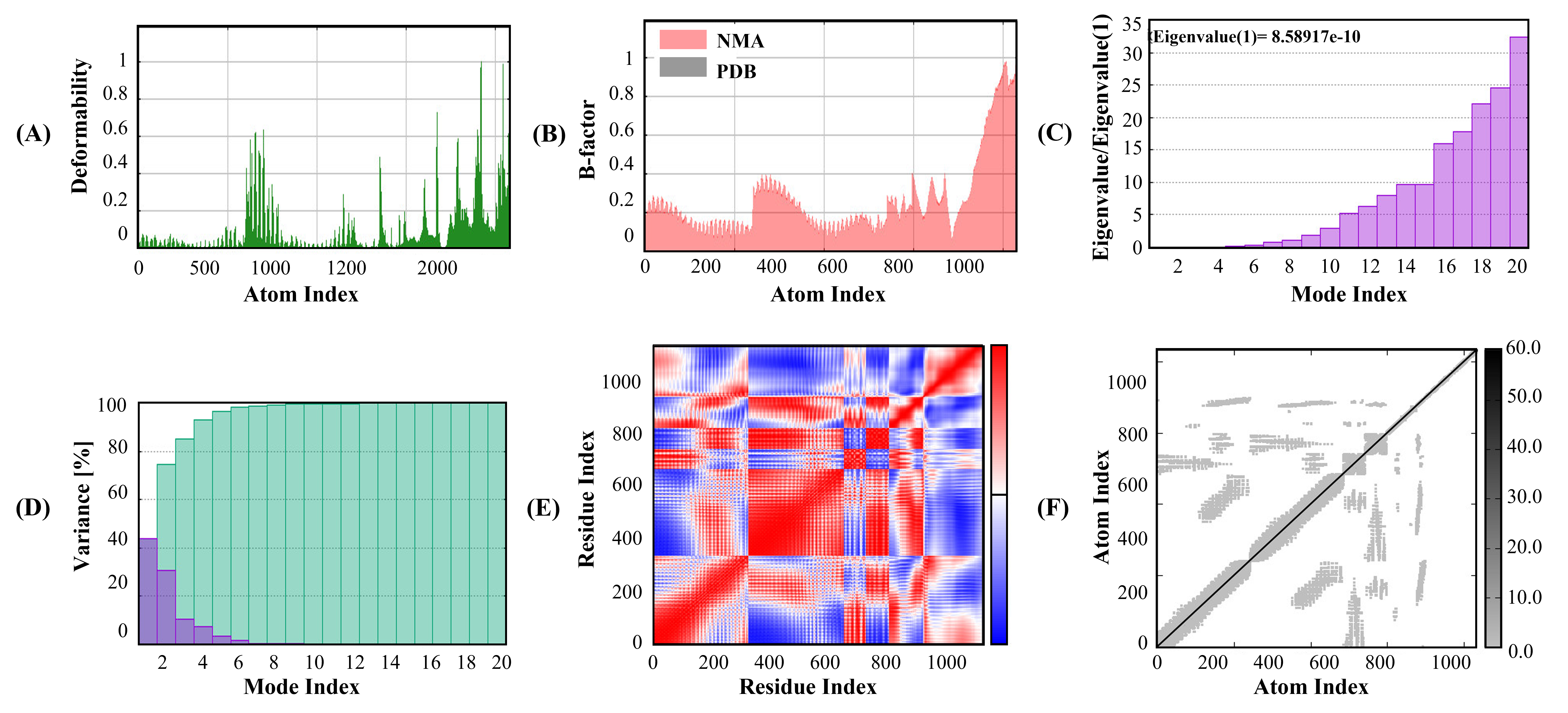

Supplement: Supplementary Figure 2 — Normal mode analysis of the V4-TLR4 docked complex (A) Deformability (B) B-factor (C) Eigenvalue- 8.589711e-10 (D) Variance (E) Covariance (F) Elastic Network. [file Image2.tif]

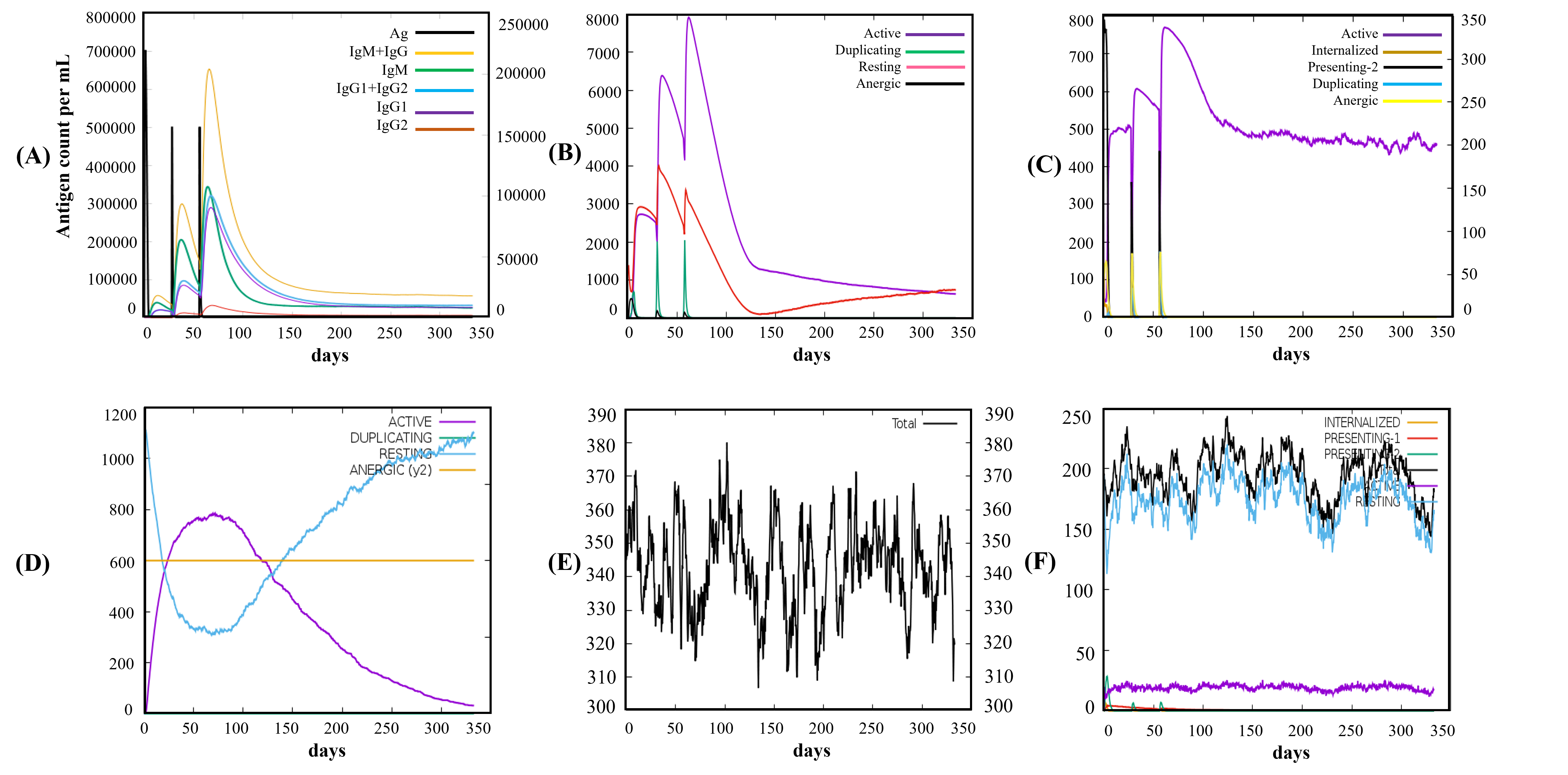

Supplement: Supplementary Figure 3 — In-silico prediction of immune simulation via the C-ImmSim server for the V2 vaccine construct. (A) IgG, IgM, IgG1, IgG2 Antibodies production (B) T helper cell population per state (C) B cell population per state (D) T cytotoxic cell population per state (E) Natural killer cell production (F) Dendritic cell population [file Image3.tif]

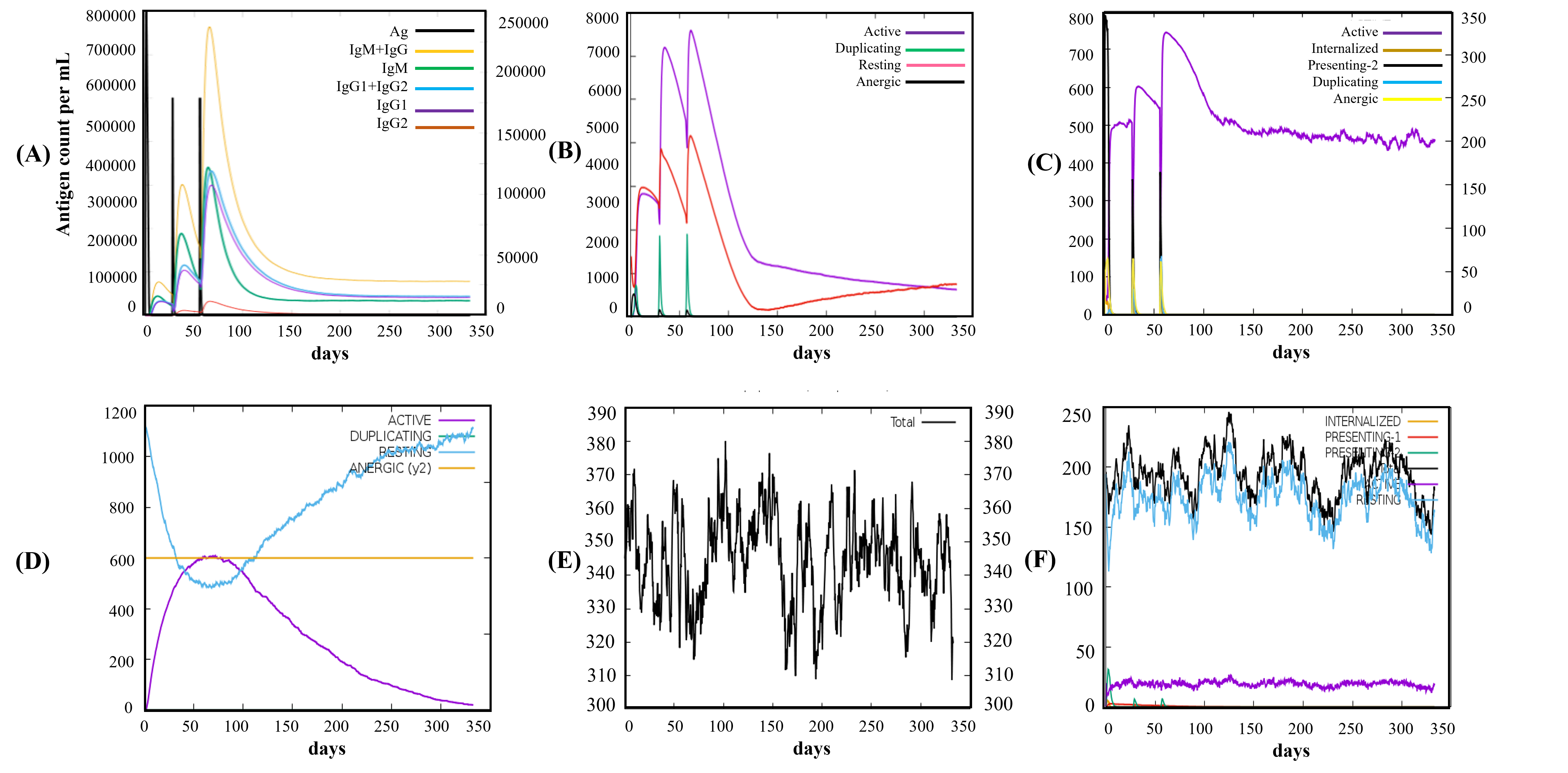

Supplement: Supplementary Figure 4 — In-silico prediction of immune simulation via the C-ImmSim server for the V4 vaccine construct. (A) IgG, IgM, IgG1, IgG2 Antibodies production (B) T helper cell population per state (C) B cell population per state (D) T cytotoxic cell population per state (E) Natural killer cell production (F) Dendritic cell population [file Image4.tif]

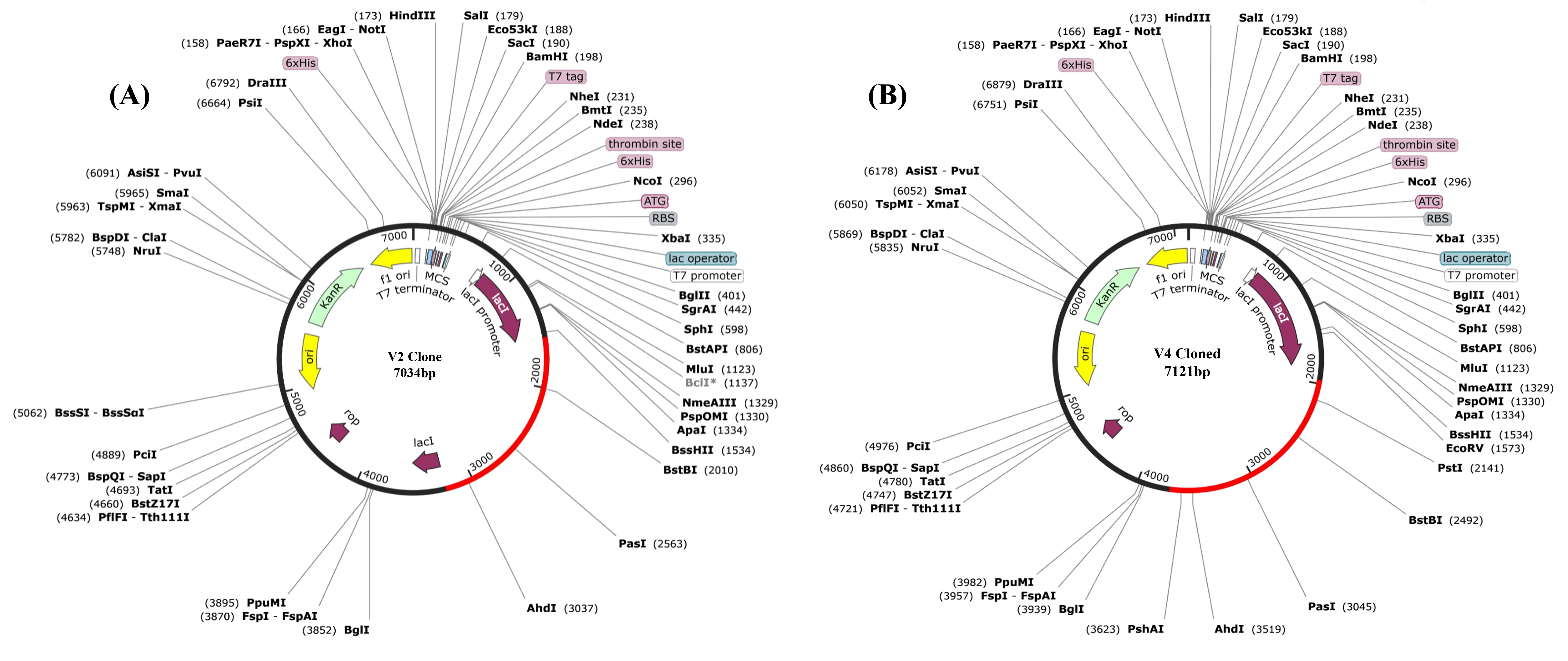

Supplement: Supplementary Figure 5 — In-silico cloning of the V2 (A) and V4 (B) into the E. coli pET28a vector. The vector, pET-28(+), was 5369 bp in length before the insertion of the chimeric vaccines, V2 and V4. (A) The total clone length of the V2 vaccine after insertion into the plasmid was 7034 bp. (B) The total clone length of the V4 vaccine after insertion into the plasmid was 7121 bp. [file Image5.tif]
