# Supplementary material for: Screening of novel therapeutic targets and chimeric vaccine construction against antibiotic-resistant Yersinia Enterocolitica
Source: Front Immunol. 2025 Jul 4;16:1555248. doi: 10.3389/fimmu.2025.1555248 (PMC12271202; doi:10.3389/fimmu.2025.1555248)
Supplement: Supplementary file 6 [file Table1.docx]

**Table S1.** Analysis of antigenicity, allergenicity, and physiochemical properties of the predicted vaccine targets

| **Protein ID** | **No. of AA** | **Topology** | **Molecular weight** | **Theoretical pi** | **Aliphatic Index** | **GRAVY value** | **Antigen** | **Allergen** | **Instability Index** |
| --- | --- | --- | --- | --- | --- | --- | --- | --- | --- |
| WP_005164390.1 | 444 | I5-27o32-54i320-351o361-383i396-418o | 50524.78 | 9.76 | 113.96 | 0.36 | Yes | No | Stable |
| WP_019079071.1 | 371 | O328-350i | 39669.14 | 6.57 | 80.78 | -0.204 | Yes | No | Unstable |
| **WP_019079224.1** | **360** | **0** | **39560.92** | **5.04** | **74.06** | **-0.419** | **Yes** | **No** | **Stable** |
| WP_019080857.1 | 692 | I21-38o42-64i76-98o113-135i209-231o251-273i294-316o | 74866.55 | 5.66 | 116.85 | 0.371 | Yes | No | Stable |
| WP_019080861.1 | 557 | O10-32i191-213o | 60424.11 | 5.11 | 91.8 | -0.158 | Yes | No | Stable |
| WP_019082060.1 | 595 | O5-27i268-290o | 61581.95 | 4.95 | 105.56 | 0.273 | Yes | No | Stable |
| WP_019083129.1 | 517 | I5-24o | 55583.97 | 4.97 | 85.01 | -0.195 | Yes | No | Stable |
| WP_019084175.1 | 640 | I21-43o53-75i88-110o137-159i172-194o214-236i243-265o270-292i313-335o | 68399.64 | 5.86 | 106.7 | 0.337 | Yes | No | Stable |
| WP_046050024.1 | 424 | 0 | 45162.15 | 5.29 | 110.94 | 0.2 | Yes | No | Stable |
| WP_046051349.1 | 552 | I13-35o | 58607.24 | 5.97 | 98.93 | -0.001 | No | No | Stable |
| WP_050131380.1 | 674 | I23-41o46-65i72-90o94-111i118-137o157-179i362-384o409-431i438-455o | 76083.69 | 9.07 | 104.23 | 0.183 | Yes | No | Unstable |
| WP_050157306.1 | 366 | 0 | 40264.98 | 4.53 | 66.39 | -0.548 | Yes | No | Stable |
| **WP_050161901.1** | **764** | **0** | **83476.04** | **5.33** | **68.97** | **-0.549** | **Yes** | **No** | **Stable** |
| WP_050163389.1 | 433 | 0 | 48110.24 | 5.42 | 77.97 | -0.464 | Yes | Yes | Stable |
| WP_263696491.1 | 249 | I19-41o69-86i215-237o | 28121.56 | 6.91 | 136.55 | 0.698 | Yes | No | Unstable |

*The rows in bold show the proteins selected for vaccine construction.
