# Supplementary material for: Screening of novel therapeutic targets and chimeric vaccine construction against antibiotic-resistant Yersinia Enterocolitica
Source: Front Immunol. 2025 Jul 4;16:1555248. doi: 10.3389/fimmu.2025.1555248 (PMC12271202; doi:10.3389/fimmu.2025.1555248)
Supplement: Supplementary file 7 [file Table2.docx]

**Table S2.** Allergenicity, antigenicity, toxicity, immunogenicity, and solubility of the MHC-I binding epitopes of protein (WP050161901.1).

| **Alleles** | **Start** | **End** | **Peptide** | **Score** | **Rank** | **Allergen** | **Antigen** | **Toxin** | **Immunogenicity** | **Water soluble** |
| --- | --- | --- | --- | --- | --- | --- | --- | --- | --- | --- |
| HLA-A*68:02 | 93 | 101 | EIIRTMPGV | 0.839614 | 0.04 | No | No | No | -0.02178 | Good |
| HLA-B*07:02 | 135 | 143 | IPVSSRNAV | 0.933055 | 0.04 | Yes | No | No | -0.24805 | Poor |
| HLA-B*51:01 | 160 | 168 | VPANMVEKI | 0.872064 | 0.02 | No | No | No | -0.14284 | Good |
| HLA-B*53:01 | 194 | 203 | QPDKELHGSW | 0.832696 | 0.03 | No | Yes | No | -0.16071 | Good |
| HLA-A*01:01 | 229 | 238 | LSDTVSFRLY | 0.977274 | 0.01 | No | Yes | No | 0.07276 | Poor |
| HLA-A*30:01 | 269 | 277 | AGREGVRNK | 0.711367 | 0.04 | Yes | Yes | No | 0.22931 | Good |
| HLA-B*40:01 | 285 | 293 | WEFAQGQSL | 0.983267 | 0.01 | Yes | Yes | No | -0.19795 | Poor |
| HLA-A*02:03 | 321 | 329 | SLYGAETNV | 0.931851 | 0.02 | Yes | Yes | No | 0.19423 | Poor |
| **HLA-B*40:01** | **392** | **400** | **SEVNLPFEL** | **0.989174** | **0.01** | **No** | **Yes** | **No** | **0.14295** | **Good** |
| HLA-A*68:01 | 463 | 471 | TTMLTPALR | 0.969864 | 0.01 | Yes | No | No | -0.01426 | Poor |
| HLA-A*30:02 | 504 | 512 | RAYKAPNLY | 0.930046 | 0.01 | No | No | No | -0.20248 | Good |
| HLA-A*01:01 | 513 | 521 | QTNPNYLLY | 0.986614 | 0.01 | No | Yes | No | -0.03888 | Poor |
| HLA-B*44:02 | 544 | 552 | AETSVNKEI | 0.916616 | 0.02 | Yes | Yes | No | -0.24326 | Good |
| HLA-B*58:01 | 593 | 601 | YANSDIFKW | 0.99581 | 0.01 | No | No | No | -0.04889 | Poor |
| HLA-B*53:01 | 618 | 626 | VPFTDTVQW | 0.989325 | 0.01 | Yes | No | No | 0.10236 | Poor |
| HLA-A*23:01 | 643 | 651 | DYLSITPEF | 0.950045 | 0.01 | Yes | Yes | No | 0.04521 | Good |
| HLA-B*57:01 | 665 | 673 | LSLLSTVTW | 0.994951 | 0.01 | Yes | No | No | -0.0818 | Poor |
| HLA-B*15:01 | 701 | 709 | AIFGLSASY | 0.939133 | 0.01 | Yes | Yes | No | -0.15807 | Poor |
| HLA-A*33:01 | 723 | 731 | NLFDKRQFR | 0.924999 | 0.01 | Yes | Yes | No | -0.13032 | Good |
| HLA-A*30:02 | 747 | 756 | ATYNEPGRTY | 0.884296 | 0.01 | Yes | Yes | No | 0.17725 | Good |

*The row in bold show the epitope selected for vaccine construction
