# Supplementary material for: Screening of novel therapeutic targets and chimeric vaccine construction against antibiotic-resistant Yersinia Enterocolitica
Source: Front Immunol. 2025 Jul 4;16:1555248. doi: 10.3389/fimmu.2025.1555248 (PMC12271202; doi:10.3389/fimmu.2025.1555248)
Supplement: Supplementary file 8 [file Table3.docx]

**Table S3.** Allergenicity, antigenicity, toxicity, immunogenicity, and solubility analysis of the MHC-1 binding epitopes of protein (WP019079224.1).

| **Alleles** | **Start** | **End** | **Peptide** | **Score** | **Rank** | **Allergen** | **Antigen** | **Toxin** | **Immunogenicity** | **Water solubility** |
| --- | --- | --- | --- | --- | --- | --- | --- | --- | --- | --- |
| HLA-B*08:01 | 1 | 9 | MMKRSVLAL | 0.886904 | 0.02 | No | No | No | -0.12666 | Good |
| HLA-A*02:03 | 14 | 22 | SMVPTLSNA | 0.793513 | 0.06 | Yes | No | No | -0.1138 | Poor |
| HLA-A*24:02 | 25 | 33 | IYNKDGNKL | 0.774851 | 0.06 | Yes | Yes | No | -0.29706 | Good |
| HLA-A*26:01 | 34 | 43 | DLYGRVAAKY | 0.596736 | 0.08 | Yes | Yes | No | 0.06735 | Good |
| HLA-B*53:01 | 50 | 58 | NADDTYVRF | 0.610783 | 0.08 | Yes | Yes | No | 0.12892 | Good |
| HLA-B*40:01 | 62 | 70 | GETQINSQL | 0.989961 | 0.01 | No | Yes | No | -0.18775 | Good |
| **HLA-A*30:01** | **94** | **103** | **KTRLGFAGLK** | **0.720608** | **0.04** | **No** | **Yes** | **No** | **0.21159** | **Good** |
| HLA-A*30:02 | 108 | 116 | GSFDYGRNY | 0.798676 | 0.02 | Yes | No | No | 0.12852 | Good |
| HLA-A*24:02 | 124 | 132 | AYTDMLPEF | 0.976828 | 0.01 | Yes | No | No | -0.09738 | Poor |
| HLA-A*26:01 | 135 | 143 | DSIAYTDNY | 0.651289 | 0.07 | Yes | No | No | 0.13045 | Good |
| HLA-A*31:01 | 147 | 155 | RSTGLATYR | 0.84841 | 0.05 | No | No | No | 0.10333 | Good |
| HLA-A*30:02 | 164 | 172 | KGLNVAAQY | 0.743584 | 0.04 | No | Yes | No | 0.03226 | Poor |
| HLA-B*15:01 | 188 | 196 | IQKANGDGY | 0.906267 | 0.02 | Yes | Yes | No | 0.03349 | Good |
| HLA-A*30:02 | 238 | 246 | QAWATALKY | 0.666187 | 0.07 | Yes | No | No | 0.05054 | Poor |
| HLA-A*68:01 | 259 | 267 | ETLNMTPYK | 0.917135 | 0.06 | No | Yes | No | -0.15609 | Good |
| HLA-A*02:03 | 269 | 277 | LIANKTQNV | 0.752488 | 0.07 | No | No | No | -0.26881 | Poor |
| HLA-B*40:01 | 285 | 293 | FENGIRPSI | 0.861464 | 0.08 | Yes | Yes | No | 0.1043 | Good |
| HLA-A*24:02 | 320 | 328 | TYYINKNMF | 0.94705 | 0.01 | Yes | No | No | -0.18464 | Poor |
| HLA-A*23:01 | 329 | 337 | TYVDYQINL | 0.79103 | 0.05 | Yes | Yes | No | 0.03162 | Poor |
| HLA-A*26:01 | 350 | 358 | DTVAVNLTY | 0.961159 | 0.01 | Yes | Yes | No | 0.1002 | Poor |

*The row in bold shows the epitope selected for vaccine construction.
