# Supplementary material for: Screening of novel therapeutic targets and chimeric vaccine construction against antibiotic-resistant Yersinia Enterocolitica
Source: Front Immunol. 2025 Jul 4;16:1555248. doi: 10.3389/fimmu.2025.1555248 (PMC12271202; doi:10.3389/fimmu.2025.1555248)
Supplement: Supplementary file 9 [file Table4.docx]

**Table S4.** Analysis of the predicted MHC-II binding peptides of proteins (WP050161901.1).

| **Allele** | **Start** | **End** | **Peptide** | **Rank** | **Allergen** | **Antigen** | **Toxin** | **IL4 inducers** | **IL10 inducers** | **IFN-gamma inducing** | **Water solubility** |
| --- | --- | --- | --- | --- | --- | --- | --- | --- | --- | --- | --- |
| HLA-DRB4*01:01 | 22 | 36 | STPLWAQTQDTTQAG | 0.43 | No | Yes | No | No | Yes | Positive | Poor |
| HLA-DRB1*15:01 | 92 | 106 | SEIIRTMPGVNLSGN | 0.64 | No | Yes | No | Yes | No | Positive | Poor |
| HLA-DRB1*03:01 | 127 | 141 | NTLIMIDGIPVSSRN | 0.61 | No | No | No | Yes | No | Positive | Poor |
| HLA-DRB1*07:01 | 226 | 240 | LGPLSDTVSFRLYGG | 0.62 | No | Yes | No | No | No | Positive | Poor |
| **HLA-DRB3*02:02** | **246** | **260** | **ADDWDINQGHESART** | **0.12** | **No** | **Yes** | **No** | **Yes** | **No** | **Yes** | **Good** |
| **HLA-DRB1*07:01** | **352** | **366** | **YVQYENTRNSRINEG** | **0.07** | **No** | **Yes** | **No** | **Yes** | **Yes** | **No** | **Good** |
| **HLA-DRB1*03:01** | **467** | **481** | **TPALRFDHHSTAGSN** | **0.48** | **No** | **Yes** | **No** | **Yes** | **No** | **Yes** | **Good** |
| HLA-DRB3*02:02 | 509 | 523 | PNLYQTNPNYLLYSR | 0.12 | No | Yes | No | No | Yes | Yes | Poor |
| HLA-DRB3*01:01 | 565 | 579 | ITYFRNDYRNKIEPG | 0.61 | No | No | No | Yes | No | Yes | Good |
| HLA-DRB1*07:01 | 700 | 714 | YAIFGLSASYTVTKN | 0.33 | No | Yes | No | Yes | No | Yes | Poor |

*The rows in bold show the selected epitopes.
