# Supplementary material for: Screening of novel therapeutic targets and chimeric vaccine construction against antibiotic-resistant Yersinia Enterocolitica
Source: Front Immunol. 2025 Jul 4;16:1555248. doi: 10.3389/fimmu.2025.1555248 (PMC12271202; doi:10.3389/fimmu.2025.1555248)
Supplement: Supplementary file 10 [file Table5.docx]

**Table S5.** Analysis of the predicted MHC-II binding peptides of protein (WP019079224.1)

| **Alleles** | **Start** | **End** | **Peptide** | **Rank** | **Allergen** | **Antigen** | **Toxin** | **IL4 inducers** | **IL10 inducers** | **IFN-gamma inducers** | **Water solubility** |
| --- | --- | --- | --- | --- | --- | --- | --- | --- | --- | --- | --- |
| HLA-DRB3*02:02 | 40 | 54 | AAKYIFTNHNNADDT | 0.3 | Yes | Yes | No | No | No | Yes | Good |
| HLA-DRB4*01:01 | 62 | 76 | GETQINSQLTGYGQW | 1.3 | No | No | No | Yes | Yes | No | Poor |
| HLA-DRB3*02:02 | 74 | 88 | GQWEYNVAAKNAESQ | 0.01 | Yes | Yes | No | Yes | No | Yes | Good |
| **HLA-DRB1*15:01** | **134** | **148** | **GDSIAYTDNYMTGRS** | **1.3** | **No** | **Yes** | **No** | **Yes** | **No** | **Yes** | **Good** |
| HLA-DRB1*07:01 | 157 | 171 | SDFFGLVKGLNVAAQ | 0.32 | No | No | No | Yes | No | Yes | Poor |
| **HLA-DRB4*01:01** | **197** | **211** | **GLSVDYQDIEGSGVG** | **0.3** | **No** | **Yes** | **No** | **Yes** | **Yes** | **Yes** | **Good** |
| HLA-DRB3*01:01 | 241 | 255 | ATALKYDANQVYIAA | 0.14 | No | No | No | Yes | No | Yes | Poor |
| HLA-DRB1*15:01 | 260 | 274 | TLNMTPYKALIANKT | 0.04 | No | No | No | Yes | Yes | Yes | Poor |
| **HLA-DRB5*01:01** | **288** | **302** | **GIRPSIAYVQSKGKD** | **0.23** | **No** | **Yes** | **No** | **Yes** | **Yes** | **Yes** | **Good** |
| HLA-DRB1*15:01 | 322 | 336 | YINKNMFTYVDYQIN | 1.4 | No | Yes | No | Yes | Yes | Yes | Poor |

*The rows in bold show the selected epitopes.
