# Supplementary material for: Screening of novel therapeutic targets and chimeric vaccine construction against antibiotic-resistant Yersinia Enterocolitica
Source: Front Immunol. 2025 Jul 4;16:1555248. doi: 10.3389/fimmu.2025.1555248 (PMC12271202; doi:10.3389/fimmu.2025.1555248)
Supplement: Supplementary file 11 [file Table6.docx]

**Table S6.** Analysis of the predicted B-cell binding epitopes of protein (WP_019079224.1).

| **Start** | **End** | **Peptide** | **Length** | **Antigenicity** | **Allergenicity** | **Toxicity** | **Water solubility** |
| --- | --- | --- | --- | --- | --- | --- | --- |
| 45 | 54 | FTNHNNADDT | 10 | Antigen | Allergen | Non-Toxin | Good |
| **63** | **72** | **ETQINSQLTG** | **10** | **Antigen** | **Non-Allergen** | **Non-Toxin** | **Good** |
| **80** | **94** | **VAAKNAESQGDKGNK** | **15** | **Antigen** | **Non-Allergen** | **Non-Toxin** | **Good** |
| **127** | **149** | **DMLPEFGGDSIAYTDNYMTGRST** | **23** | **Antigen** | **Non-Allergen** | **Non-Toxin** | **Good** |
| 173 | 195 | QGRNDDGDTTKNERAIQKANGDG | 23 | Antigen | Allergen | Non-Toxin | Good |
| **217** | **238** | **SNSNRTLGQKNLANSATGDKAQ** | **22** | **Antigen** | **Non-Allergen** | **Non-Toxin** | **Good** |
| 259 | 275 | ETLNMTPYKALIANKTQ | 17 | Non-Antigen | Non-Allergen | Non-Toxin | Good |
| 299 | 311 | KGKDLDAVGSADL | 13 | Antigen | Allergen | Non-Toxin | Good |
| 336 | 349 | NLLDENNPLGLGTD | 14 | Non-Antigen | Non-Allergen | Non-Toxin | Good |

*The rows in bold show the selected epitopes
