# Supplementary material for: Screening of novel therapeutic targets and chimeric vaccine construction against antibiotic-resistant Yersinia Enterocolitica
Source: Front Immunol. 2025 Jul 4;16:1555248. doi: 10.3389/fimmu.2025.1555248 (PMC12271202; doi:10.3389/fimmu.2025.1555248)
Supplement: Supplementary file 12 [file Table7.docx]

**Table S7.** Analysis of the predicted B-cell binding epitopes of protein (WP_050161901.1).

| **Start** | **End** | **Peptide** | **Length** | **Antigenicity** | **Allergenicity** | **Toxicity** | **Water solubility** |
| --- | --- | --- | --- | --- | --- | --- | --- |
| 29 | 58 | TQDTTQAGSDTAATQQKETTATDNKATTGD | 30 | Antigen | Allergen | Non-Toxin | Good |
| **63** | **89** | **TAQQQVRQALGASTITAEDIRKRPPAN** | **27** | **Antigen** | **Non-Allergen** | **Non-Toxin** | **Good** |
| **104** | **115** | **SGNSASGQRGNN** | **12** | **Antigen** | **Non-Allergen** | **Non-Toxin** | **Good** |
| 139 | 162 | SRNAVRYGWRGERDTRGDTNWVPA | 24 | Antigen | Allergen | Non-Toxin | Good |
| 195 | 204 | PDKELHGSWN | 10 | Antigen | Allergen | Non-Toxin | Good |
| **209** | **222** | **LPQHSEEGATRRTD** | **14** | **Antigen** | **Non-Allergen** | **Non-Toxin** | **Good** |
| **241** | **276** | **YNKTDADDWDINQGHESARTGNQAGTLPAGREGVRN** | **36** | **Antigen** | **Non-Allergen** | **Non-Toxin** | **Good** |
| 299 | 333 | YSRQGNIYAGDTQNTNSNAIVRSLYGAETNVMYRD | 35 | Antigen | Allergen | Non-Toxin | Good |
| 336 | 350 | SLTHRGFWDNGVSTT | 15 | Non-Antigen | Allergen | Non-Toxin | Poor |
| **354** | **383** | **QYENTRNSRINEGLAGGTEGIFSNNYFSTI** | **30** | **Antigen** | **Non-Allergen** | **Non-Toxin** | **Good** |
| 413 | 446 | NDQKMNDPTSNTQTTTEGGSVSGLTGTGRNTRTS | 34 | Antigen | Allergen | Non-Toxin | Good |
| 509 | 544 | PNLYQTNPNYLLYSRGQGCYGGGGSCYLMGNDDLSA | 36 | Antigen | Allergen | Non-Toxin | Poor |
| 578 | 602 | PGLVSLGTASGGTGTYANSDIFKWE | 25 | Antigen | Non-Allergen | Non-Toxin | Poor |
| **634** | **650** | **LESKNKSTGDYLSITPE** | **17** | **Antigen** | **Non-Allergen** | **Non-Toxin** | **Good** |
| **675** | **697** | **GRQKPKKYDYQGLPVTGTARNEV** | **23** | **Antigen** | **Non-Allergen** | **Non-Toxin** | **Good** |
| **726** | **754** | **DKRQFREGNAQNVANIAGAGAATYNEPGR** | **29** | **Antigen** | **Non-Allergen** | **Non-Toxin** | **Good** |

*The rows in bold show the selected epitopes
