# Supplementary material for: Screening of novel therapeutic targets and chimeric vaccine construction against antibiotic-resistant Yersinia Enterocolitica
Source: Front Immunol. 2025 Jul 4;16:1555248. doi: 10.3389/fimmu.2025.1555248 (PMC12271202; doi:10.3389/fimmu.2025.1555248)
Supplement: Supplementary file 13 [file Table8.docx]

| **Table S8.** Screening of the antigenicity, allergenicity, solubility, toxicity, and physicochemical properties of the vaccine constructs. | | | | | | | | | | | |
| --- | --- | --- | --- | --- | --- | --- | --- | --- | --- | --- | --- |
| **Vaccine construct** | **SolPro** | **AntigenPro** | **VaxiJen** | **Allergen** | **M. wt** | **Theoretical pi** | **GRAVY value** | **Aliphatic index** | **Instability index** | **Amino acids** | **Topology** |
| V1 | 0.93636 | 0.92732 | Antigen | No | 51309.8 | 9.79 | -1.12 | 48.39 | [36.12] stable | 479 | 0 |
| V2 | 0.8749 | 0.94617 | Antigen | No | 58640.9 | 9.28 | -0.906 | 59.57 | [31.91] stable | 555 | 0 |
| V3 | 0.60498 | 0.93676 | Antigen | No | 70567.5 | 9.61 | -0.897 | 63.94 | [37.71] stable | 662 | 0 |
| V4 | 0.89833 | 0.94595 | Antigen | No | 62659 | 9.14 | -0.976 | 59.79 | [39.56] stable | 584 | 0 |
