# Supplementary material for: Screening of novel therapeutic targets and chimeric vaccine construction against antibiotic-resistant Yersinia Enterocolitica
Source: Front Immunol. 2025 Jul 4;16:1555248. doi: 10.3389/fimmu.2025.1555248 (PMC12271202; doi:10.3389/fimmu.2025.1555248)
Supplement: Supplementary file 14 [file Table9.docx]

**Table S9.** Conformational B-cell-forming residues identified in the Vaccine-2.

| **Sr. No.** | **Residues** | **No of residues** | **Score** |
| --- | --- | --- | --- |
| 1 | A:K521, A:D522, A:K523, A:R524, A:Q525, A:F526, A:R527, A:E528, A:G529, A:N530, A:A531, A:Q532, A:N533, A:V534, A:A535, A:N536, A:I537, A:A538, A:G539, A:A540, A:G541, A:A542, A:A543, A:T544, A:Y545, A:N546, A:E547, A:P548, A:G549, A:R550, A:E551, A:A552, A:A553, A:A554, A:K555 | 35 | 0.967 |
| 2 | A: R516, A:E518, A:V519, A:K520 | 4 | 0.932 |
| 3 | A: D505, A:Y506, A:Q507, A:G508, A:L509, A:P510, A:V511, A:T512, A:G513, A:T514, A:A515 | 11 | 0.914 |
| 4 | A:K298, A:K299, A:D300, A:M301, A:L302, A:P303, A:E304, A:F305, A:G306, A:G307, A:D308, A:S309, A:I310, A:A311, A:Y312 | 15 | 0.885 |
| 5 | A:K502, A:K503, A:Y504 | 3 | 0.857 |
| 6 | A:K282, A:V283, A:A284, A:A285, A:K286, A:N287, A:A288, A:E289, A:S290, A:Q291, A:G292, A:D293, A:K294, A:G295, A:N296 | 15 | 0.857 |
| 7 | A:Q499, A:K500, A:P501 | 3 | 0.784 |
| 8 | A:T313, A:D314, A:N315, A:Y316, A:M317, A:T318, A:G319, A:R320, A:S321, A:T322, A:K323 | 11 | 0.741 |
| 9 | A:M1, A:S2, A:D3, A:L4, A:K5, A:N6, A:L7, A:A8, A:E9, A:T10, A:L11, A:V12, A:N13, A:L14, A:T15, A:V16, A:K17, A:D18, A:V19, A:N20, A:E21, A:L22, A:A23, A:A24, A:I25, A:L26, A:K27, A:D28, A:E29, A:Y30, A:G31, A:I32 | 32 | 0.725 |
| 10 | A:S263, A:T264, A:A265, A:G266, A:S267, A:N268, A:K269, A:K270, A:E271, A:T272, A:Q273, A:I274, A:N275, A:S276, A:Q277, A:L278, A:T279, A:G280, A:K281 | 19 | 0.718 |
| 11 | A:K391, A:L392, A:P393, A:Q394, A:H395, A:S396, A:E397, A:E398, A:G399, A:A400, A:T401, A:R402 | 12 | 0.683 |
| 12 | A:L59, A:K60, A:S61, A:A62, A:G63, A:A64, A:S65, A:K66, A:L67, A:A68, A:V69, A:V70, A:K71, A:L72, A:V73, A:K74, A:D75, A:L76, A:T77, A:G78, A:A79, A:G80, A:L81, A:K82, A:E83, A:A84, A:K85, A:D86, A:M87, A:V88, A:D89, A:G90, A:A91, A:P92, A:A93, A:A94, A:I95, A:K96, A:I99, A:S100, A:D102, A:E103, A:E105, A:A106, A:L107, A:K108, A:K109, A:Q110, A:L111, A:E112, A:A114, A:G115, A:A116, A:E117, A:V118 | 55 | 0.632 |
| 13 | A:A360, A:S361, A:T362, A:I363, A:T364, A:A365, A:E366, A:D367, A:I368, A:R369, A:K370, A:R371, A:P372, A:P373, A:A374, A:N375, A:K376, A:K377, A:S378, A:G379, A:N380, A:S381, A:Q385, A:R386, A:G387, A:N388, A:N389, A:K390, A:R403, A:T404, A:D405, A:K406, A:K407, A:Y408, A:N409 | 35 | 0.623 |
| 14 | A:K495, A:G497, A:R498 | 3 | 0.592 |
| 15 | A:E33, A:P34, A:A35, A:A36, A:A37, A:A38, A:V39, A:M41 | 8 | 0.584 |
| 16 | A:R187, A:S188, A:G189, A:P190, A:G191, A:P192, A:G193, A:G194, A:L195, A:S196, A:V197, A:D198, A:Y199 | 13 | 0.569 |
| 17 | A:K410, A:T411, A:D412, A:A413, A:D414, A:D417, A:I418 | 7 | 0.551 |
