# Supplementary material for: Screening of novel therapeutic targets and chimeric vaccine construction against antibiotic-resistant Yersinia Enterocolitica
Source: Front Immunol. 2025 Jul 4;16:1555248. doi: 10.3389/fimmu.2025.1555248 (PMC12271202; doi:10.3389/fimmu.2025.1555248)
Supplement: Supplementary file 15 [file Table10.docx]

**Table S10.** Conformational B-cell-forming residues were identified in the Vaccine-4.

| **Sr. No.** | **Residues** | **No of residues** | **Score** |
| --- | --- | --- | --- |
| 1 | A:T277, A:G278, A:P279, A:G280, A:P281, A:G282, A:T283, A:P284, A:A285, A:L286, A:R287, A:F288, A:D289, A:H290 | 14 | 0.953 |
| 2 | A:L518, A:S519, A:I520, A:T521, A:P522, A:E523 | 6 | 0.888 |
| 3 | A:M1, A:A2, A:E3, A:N4, A:S5, A:N6, A:I7, A:D8, A:D9, A:I10, A:K11, A:A12, A:P13, A:L14, A:L15, A:A16, A:A17, A:L18, A:G19, A:A20, A:A21, A:D22, A:L23, A:A24, A:L25, A:A26, A:T27, A:V28, A:N29, A:E30, A:L31, A:I32, A:T33, A:N34, A:L35, A:R36, A:E37, A:R38 | 38 | 0.85 |
| 4 | A:D443, A:D444, A:W445, A:D446, A:I447, A:N448, A:Q449, A:G450, A:H451, A:E452, A:S453, A:A454, A:R455, A:T456, A:G457, A:N458, A:Q459, A:A460, A:G461, A:T462, A:L463, A:P464, A:A465, A:G466, A:R467, A:E468, A:G469, A:V470, A:R471, A:N472, A:K473, A:K474, A:Q475, A:Y476, A:E477, A:N478, A:T479, A:R480, A:N481, A:S482, A:R483, A:I484, A:N485, A:E486, A:G487, A:L488, A:A489, A:G490, A:G491, A:T492, A:E493, A:G494, A:I495, A:F496, A:S497, A:N498, A:N499, A:Y500, A:F501, A:S502, A:T503, A:I504, A:K505, A:K506, A:L507, A:E508, A:S509, A:K510, A:N511, A:K512, A:S513, A:T514, A:G515, A:D516, A:Y517 | 75 | 0.828 |
| 5 | A:K524, A:G526, A:R527 | 3 | 0.808 |
| 6 | A:R253, A:I254, A:N255, A:E256, A:G257, A:G258, A:P259, A:G260, A:P261, A:G262, A:A263, A:D264, A:D265, A:W266, A:D267, A:I268, A:N269, A:Q270, A:G271, A:H272, A:E273, A:S274, A:A275, A:R276 | 24 | 0.773 |
| 7 | A:H291, A:S292, A:T293, A:A294, A:G295, A:S296, A:N297, A:K298, A:K299, A:E300, A:T301, A:Q302, A:I303, A:N304, A:S305, A:Q306, A:L307, A:T308, A:G309, A:K310, A:V312, A:A313, A:A314, A:K315, A:N316, A:A317, A:E318, A:S319, A:Q320, A:G321, A:D322, A:K323, A:G324, A:N325, A:K326, A:K327, A:K328, A:D329, A:M330, A:L331, A:P332, A:E333, A:F334, A:G335, A:G336, A:D337, A:S338, A:I339, A:A340, A:Y341 | 50 | 0.717 |
| 8 | A:A98, A:A99, A:L100, A:E101, A:R102, A:L103, A:R104, A:S105, A:Q106, A:Q107, A:S108, A:F109, A:E110, A:E111, A:V112, A:S113, A:A114, A:R115, A:E117, A:G118 | 20 | 0.591 |
| 9 | A:T342, A:D343, A:N344, A:Y345, A:M346, A:T347, A:G348, A:R349, A:S350, A:T351, A:K352, A:K353, A:S354, A:N355, A:S356, A:N357, A:R358, A:L360, A:G361, A:N364, A:L365, A:S368 | 22 | 0.585 |
| 10 | A:N575, A:E576, A:P577, A:G578, A:E580, A:A581, A:A582, A:A583, A:K584 | 9 | 0.551 |
| 11 | A:K531, A:K532, A:Y533, A:D534, A:Y535, A:Q536, A:L538 | 7 | 0.546 |
| 12 | A:A39, A:E40, A:E41, A:T42, A:R43, A:R46 | 6 | 0.542 |
| 13 | A:G371, A:D372, A:Q375, A:K376 | 4 | 0.541 |
| 14 | A:A379, A:Q380, A:Q382, A:V383, A:R384, A:A386, A:L387, A:G388, A:A389, A:S390 | 10 | 0.538 |
